# Plasma Activate Nitrogen for Efficient Ammonia Synthesis: Plasma Ammonia Synthesis

**DOI:** 10.1002/EXP.20240438

**Published:** 2026-02-05

**Authors:** Yamei Zhang, Jianfei Yu, Haixia Wu, Zhijun Zhao, Hongjin Xia, Qingyang Li, Yiran Wang, Dongyang Li, Qian Wang

**Affiliations:** ^1^ School of Chemical and Pharmaceutical Engineering Hebei University of Science and Technology Shijiazhuang China; ^2^ College of New Materials and Chemical Engineering Beijing Institute of Petrochemical Technology Beijing China; ^3^ Institute of Advanced Wear & Corrosion Resistant and Functional Materials Jinan University Guangzhou China; ^4^ Institute of Process Engineering Chinese Academy of Sciences Beijing China; ^5^ Department of Chemical and Materials Engineering University of Alberta Edmonton Canada

**Keywords:** ammonia, catalysis, electrochemistry, energy conversion, nitrogen chemistry, plasma

## Abstract

Synthetic ammonia is of great significance to the development of industry and agriculture. At present, its manufacturing production is still dominated by century‐old Haber–Bosch process, involving in huge amounts of energy consumptions and carbon dioxide emissions. Hence, it is of great significance to develop green and sustainable alternative technologies, such as photo‐, thermo‐, and electro‐catalytic syntheses of ammonia. Nevertheless, the intrinsic inertness of nitrogen results in low ammonia yield of above candidates, which still cannot meet commercialization expectations. Recent researches have successfully demonstrated that plasma can effectively activate the decomposition of nitrogen and hydrogen under ambient conditions, thereby significantly improving the ammonia yield of these alternative strategies. This review systematically summarizes the classification and reaction mechanism of plasma for this reason, and discusses the synergy between different plasma and catalytic methods along with energy consumptions in detail. In the meantime, the future development trend is also forecasted.

## Graphical abstract

This review systematically summarizes the classification and reaction mechanism of plasma, and discusses the synergy between different plasmas and catalytic methods along with energy consumptions in detail, in order to gain a deeper understanding of currently ammonia synthesis strategies. Their development trends are also forecasted for replacing the Haber–Bosch process.

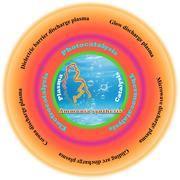

## 1 Introduction

Ammonia (NH_3_) is an indispensable raw material for industrial and agricultural productions and meanwhile, it is an outstanding hydrogen (H_2_) energy carrier with the advantages of high energy density (∼4.25 kWh L^−1^), convenient transportation and storage [1, 2, 3, 4, 5]. Up to now, the synthetic NH_3_ industry is still dominated by the century‐old Haber–Bosch (H‐B) process (over 90% of annual global production), in which the reaction needs very harsh temperature (350–450°C) and pressure (100–200 bar) along with the catalysts [6], involving in large amounts of energy consumptions (1%–2% of the world's energy sources) as well as carbon dioxide emissions (∼1.3%) [7]. Among them, most of the energy consumptions are spent on the dissociation of triple bond (N≡N), which is recognized as a critical step for deriving the nitrogen (N_2_) reduction reaction (NRR, N_2_ + 3H_2_ → 2NH_3_) [8]. How to boost N_2_ activation under mild conditions is therefore of great significance to energy conservation, emission reduction, and sustainable NH_3_ synthesis.

N_2_ is characterized by high bond dissociation enthalpy (941 kJ mol^−1^), non‐polarity, large ionization potential, negative electron affinity energy, and broad gap between the lowest unoccupied molecular orbital and the highest occupied molecular orbital (∼10.82 eV), and thus its activation is extremely difficult [9, 10, 11]. Although high temperature is conducive to the dissociation of N≡N bond and the desorption of intermediates on the catalyst surface, excessive temperatures are thermodynamically unfavorable toward NH_3_ synthesis, because the NRR is essentially exothermic reaction (ΔH = 46.27 kJ mol^−1^). Obviously, raising the NRR temperature is in conflict with N_2_ activation. This requires the exploration of more mild alternative technologies for realizing green and efficient N_2_ activation, which is an urgent challenge to be met by synthetic NH_3_ industry, currently [12, 13].

**FIGURE 1:**
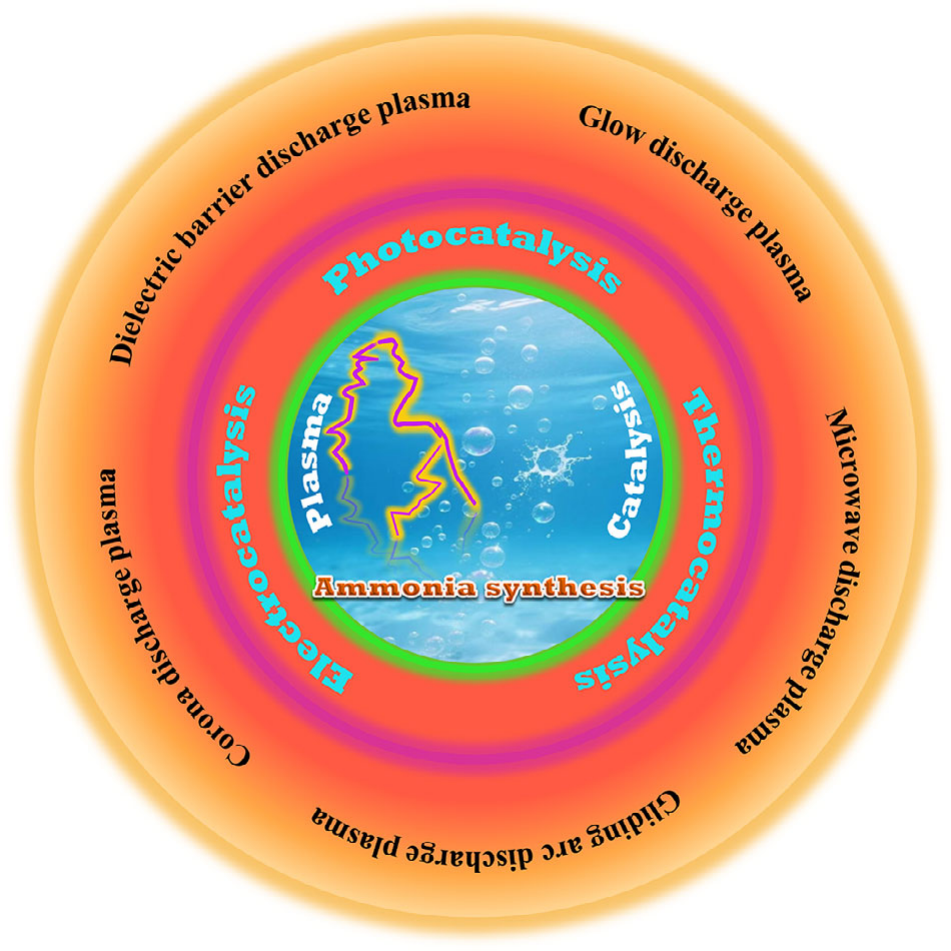
Synergistic effect between plasma and catalysis on NH_3_ synthesis.

So far, both microelectronic and macroscopic fields have been demonstrated to be effective in activating N_2_. For the microelectronic effect, it is mainly owing to the catalyst modification, for example, defect engineering, crystal facet tailoring, and hybrid strategy [14, 15, 16, 17, 18, 19, 20]. In this regard, scholars have devoted considerable efforts to activating N_2_ through catalyst design [21]. For example, introducing defect (i.e., vacancy and heteroatom) into the catalyst at the atomic level can effectively manipulate its electronic structure, thereby improving the chemical affinity of catalytically active center to N_2_. Tailoring the atomic arrangement of catalyst, including alloying, amorphization, crystal engineering and control, etc., may also significantly activate N_2_. Besides, the producing hybrid catalyst through combined different components and structural heterogeneities is feasible method for enhancing the chemisorption and activation of N_2_ [22, 23, 24, 25]. As for the macroscopic effect, it mainly includes plasma, light and electric fields. Among them, the plasma field is used to trigger the discharge of N_2_ molecules, thereby producing excited or sub‐stable species, and achieving their activation. The light field can excite the catalyst to generate photo‐generated electrons, hot electrons, and photo‐thermal effects, which may promote the adsorption and activation processes of N_2_. The electric field usually possesses highly localized electron density along with beneficial polarization effect on the N_2_, which boost the charge transfer from catalyst to N_2_, thus significantly reducing the energy level in the lowest unoccupied molecular orbital of N_2_. Although the catalyst can optimize the electronic structure of active sites through defect engineering and crystal surface regulation, etc., thereby promoting the N_2_ affinity, the improvement effect is limited by its intrinsic properties. It is therefore difficult to achieve the efficient dissociation of N_2_ under mild conditions. As for the light field, it majorly depends on the generation efficiency of photo‐generated carriers. The polarization effect of electric field may also decrease the lowest orbital energy level of N_2_, but its scope of work is highly localized. In contrast, the plasma‐activated N_2_ possesses relatively unique advantages, such as efficient energy transfer and mild reaction condition. Moreover, it circumvents the linear scaling relationship between the adsorption energy of nitrogen species and the dissociation energy of N_2_ in conventional thermocatalysis, opening up new reaction pathways on the catalyst surface. It can also be coupled with electrical, optical, and thermal catalysis to form multi‐field synergy, thereby realizing the energy consumption optimization. Recent advances in activation of N_2_ with light field [26, 27], electric field [28, 29, 30], and plasma field [31, 32, 33], have been summarized by researchers in detail. However, most of them mainly focus on a single field [34, 35, 36], ignore the cascade design with other technologies, especially the synergistic effect of plasma and catalysis on the N_2_ activation, and fail to quantitatively analyze their energy consumptions. This is the motivation for writing this review on the plasma activation of N_2_ for efficient NH_3_ synthesis. The review wants to put forward some suggestions for the future development direction of plasma‐catalytic NH_3_ synthesis, through systematically summarizing the current status at domestic and international researches. For instance, the influences of different plasmas and catalytic systems on the NH_3_ synthesis are systematically summarized, and they are classified and analyzed in detail, aiming at the synergistic effect of plasma catalysis, as schematically illustrated in Figure 1.

## 2 Plasma‐Enhanced NH_3_ Synthesis

### 2.1 Plasma‐Enhanced NH_3_ Synthesis Processes

The characteristics of plasma are mainly determined by its primitive reactions, involving in the ionization, excitation and dissociation, composition, attachment and detachment.

**FIGURE 2:**
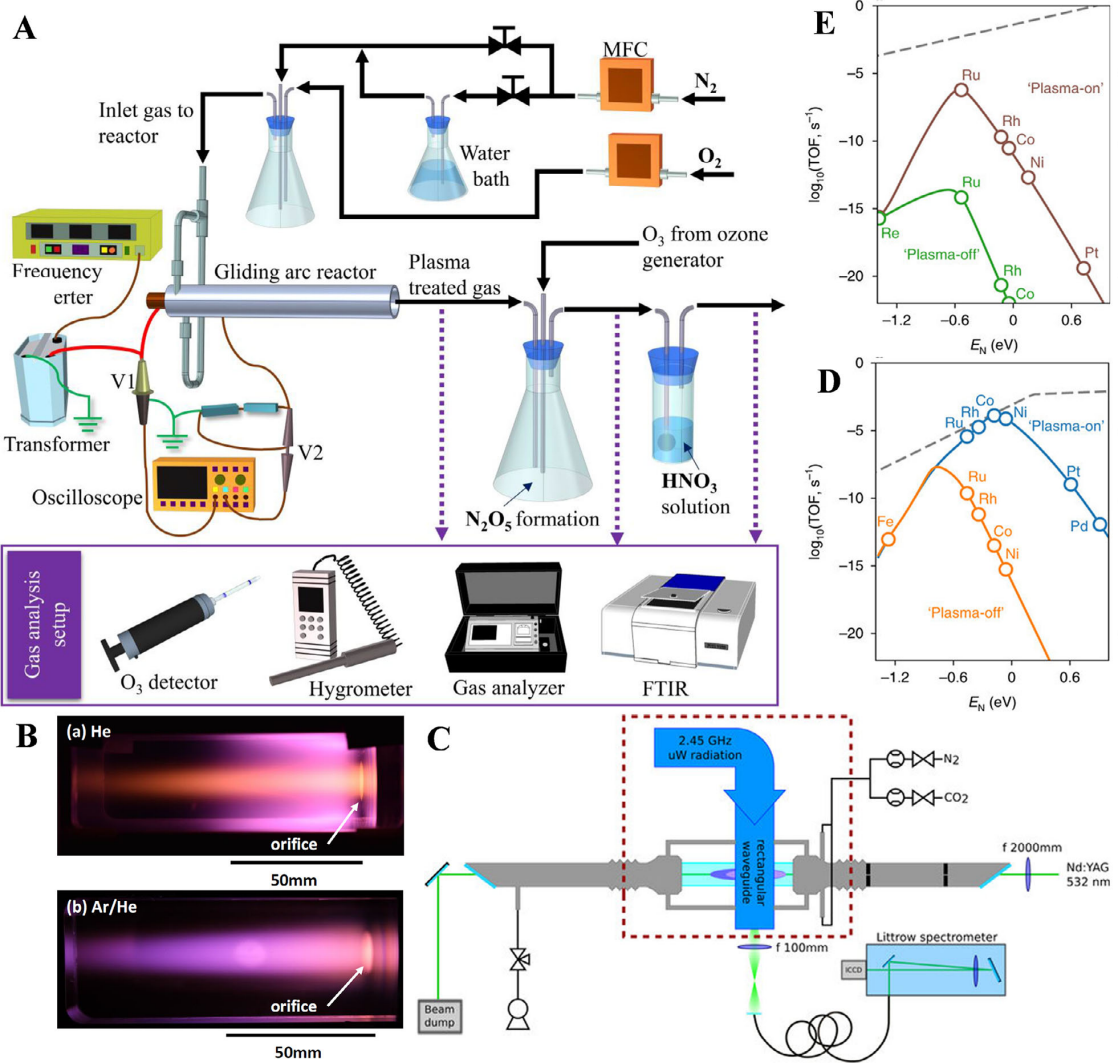
(A) Schematic diagram of the GAD reactor. Reproduced with permission Ref. [38]. (B) Photographs of the He and Ar‐based plasmas. Reproduced with permission Ref. [37]. (C) Schematic of the spontaneous Raman scattering setup installed at the flowing MW plasma reactor. Reproduced with permission Ref. [41]. (D) Comparison of N_2_ vibrational‐distribution‐weighted and thermal NH_3_ synthesis rates on the step, and (E) terrace sites, respectively. Reproduced with permission Ref. [42].

1. Ionization: The process of neutral atoms or molecules losing electrons and forming cations is called ionization, which is the first and most central process in the plasma chemistry. Electrons first obtain energy under the action of electric field, and offer energy to other components, resulting in the ionization, excitation, dissociation and other plasma chemical processes, due to their low mass and high mobility. The ionization process can be mainly divided into direct electron collisional ionization, electron‐collisional accumulated ionization, heavy‐particle collisional ionization, photoionization, surface ionization and electron emission. The ground state electrons may separate from the atoms, thereby triggering the generation of a series of charged particles, through the ionization process.
2. Excitation and dissociation: The high chemical activity of plasma is based on its super‐equilibrium concentration of active particles, including excited state particles [37]. The collision between neutral particles and electrons causes electron transitions to form excited state particles. In this connection, Denra et al. [38]. studied the N_2_ fixation in the plasma at surrounding conditions (Figure 2A), and proposed that there were three excitation modes, including rotational excitation, vibrational excitation, and electronic excitation. Among them, the excitation degree can be expressed by rotation, vibration, and electronic excitation temperature, while the electronic excitation temperature is comparable to electron temperature, and the gas temperature, ion temperature, and rotational temperature are also equal to each other [39]. However, the vibrational temperature may rise above the gas temperature in the plasma. The excitation process of plasma is often accompanied by the decomposition of substances and the generation of reactive molecules, so it plays a key role in the plasma chemical reaction and substance synthesis. The vibration excitation energy of N_2_ is 0.29 eV, and the electronic excitation energy is 6.17 eV. Both of them are lower than the dissociation energy of N_2_. At the same time, the vibration excitation has also a lower energy threshold, compared with that of electronic excitation energy. As a result, the vibration excitation may be the main excitation mode of N_2_ in the atmospheric pressure plasma. And most of the electron‐molecular exchange energy will be used for vibration excitation [40].

Yamazaki and Sasaki [37] comparatively investigated the vibrational temperature of N_2_ in the downstream of helicon‐wave excited helium (He) and argon (Ar)‐based plasmas, respectively. As shown in Figure 2B, the vibrational temperature in the He plasma is higher than that in the ionizing Ar/He plasma. In general, a higher vibrational temperature is more beneficial to boost the reactions powered by vibrational excited states. Gatti et al. [41]. also assessed the preferential vibrational excitation of N_2_ in the MW plasma by Raman scattering (Figure 2C), and confirmed that the vibrational excitation is promising strategy for promoting the chemical reactivity of endothermic reactions. Moreover, Mehta et al. [42]. developed a micro‐kinetic model incorporating the potential effects of vibrational excitation in N_2_, based on the density functional theory (DFT) calculations reported in the literature, and proposed that the transition metals bound to N_2_ were too weak to be catalytically active in the thermal reactions. The reaction rate can be increased more efficiently in the plasma catalytic reaction (Figure 2D), and the vibrational excitation of N_2_ can increase the synthesis rate of NH_3_ by reducing the dissociative adsorption energy (Figure 2E). These results provide an important guidance for optimizing catalysts of the plasma‐assisted NH_3_ synthesis. In addition to excitation, the collisions between electrons and neutral particles can lead to molecular dissociation, while the binding energy of the molecule‐ion composition may also cause the intermediates to dissociate and excite the dissociated products.

1. Composition: The process of positive and negative charged particles produced by the ionization recombine into neutral particles is called composition, which can be divided into spatial and surface compositions, depending on the location where it occurs. The composition is regarded as the reverse process of ionization, and usually excites the composite product as well.
2. Attachment and detachment: The process of atoms or molecules adsorbing electrons to generate negative ions is called attachment. On the contrary, the process of desorbing electrons to produce atoms and molecules is called detachment. Understanding the above processes is very important to investigate the mechanism responsible for plasma‐enhanced NH_3_ synthesis.

### 2.2 Plasma‐Enhanced Mechanism

**FIGURE 3:**
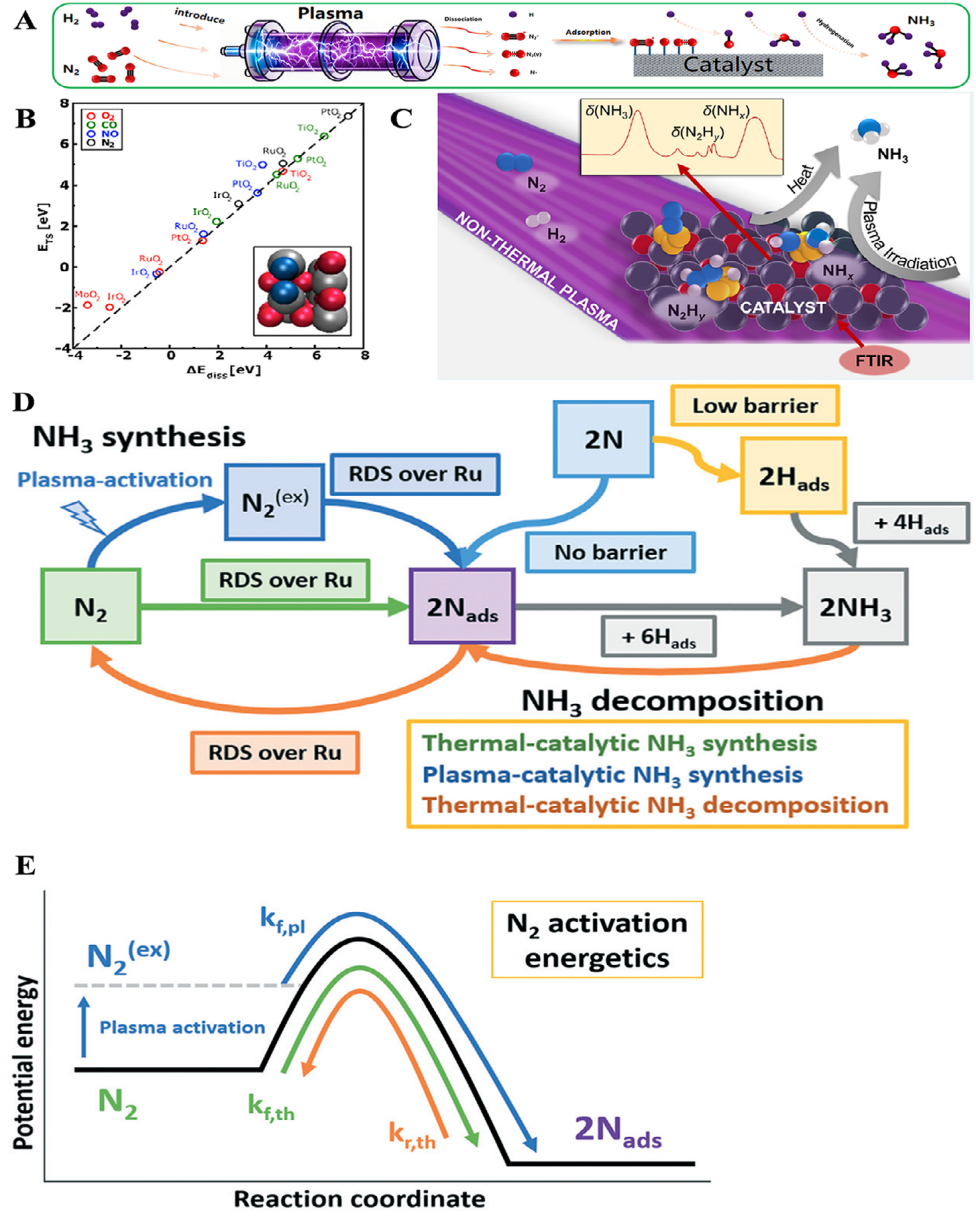
(A) Mechanism diagram of plasma‐enhanced NH_3_ synthesis. (B) Calculated transition state energies versus dissociative chemisorption energies for N_2_, CO, O_2_, and NO on the (110) stoichiometric surface of rutile oxides. Reproduced with permission Ref. [43]. (C) Identifying surface reaction intermediates in plasma catalytic NH_3_ synthesis via the FTIR spectroscopy. Reproduced with permission Ref. [44]. (D) Schematic representation, and (E) free energy for the thermal‐catalytic, plasma‐catalytic NH_3_ synthesis and thermal‐catalytic NH_3_ decomposition. Reproduced with permission Ref. [45].

Understanding the mechanism of plasma‐activated N_2_ process is of great significance for enhancing the NH_3_ synthesis. As illustrated in Figure 3A, the enhancement process involves two key stages, that is, the plasma activation of N_2_ and H_2_ to generate active species, followed by the combination of these active species to form NH_3_. Specifically, the activation of N_2_ and H_2_ occurs within the plasma, producing active particles such as nitrogen molecule (N_2_^*^), vibrationally excited state of nitrogen [N_2_ (v)], nitrogen radical (N•), and hydrogen atom (H). These particles subsequently participate in catalytic reactions on the catalyst surface. The N_2_^*^ are excited to produce N_2_ (v) through electron collisions. This process is primarily characterized by vibrational and electronic excitation. The plasma‐activated N_2_ (v) can then adsorb onto the catalyst surface, and dissociate into the N•. Similarly, the activation process of H_2_ under plasma action is also the H_2_ dissociates into H atoms. The active species are combined to synthesize NH_3_ by a stepwise hydrogenation process of N_2_. Among them, the NH• radicals are mainly generated through the combination of active nitrogen species and active hydrogen species on the catalyst surface. The NH• intermediates can further undergo hydrogenation with active hydrogen species to form NH_2_• radicals, and eventually produce NH_3_. In summary, the active particles generated by plasma under relatively mild conditions exhibit high reactivity, which effectively break the N≡N bond of N_2_ molecules and the strong chemical bonds of H_2_ molecules, thereby promoting NH_3_ generation.

Based on above considerations, it can be concluded that the plasma‐catalyzed NH_3_ synthesis can circumvent many potential limitations, compared with conventional thermocatalysis. For example, although a high temperature provides energy for N_2_ dissociation during the thermocatalysis reaction process, NH_3_ only exhibits thermodynamic activity under low temperature. Therefore, it is necessary to simultaneously apply high pressure under high temperature conditions to ensure the reaction rate, thereby shifting the equilibrium reaction to the product. For this reason, Vojvodic et al. [43]. screened the catalysts required for the low‐pressure and low‐temperature NH_3_ synthesis, and defined an ideal scaling relation between the activation energy for N_2_ dissociation and the N adsorption energy for characterizing active catalyst, as presented in Figure 3B. Furthermore, the thermocatalysis NH_3_ synthesis is limited by the linear relationship between the binding strength of catalyst surface to N‐containing species and the N_2_ dissociation. On the other hand, the molecular vibration, electron excitation, and electron impact dissociation induced by the high‐energy electrons overcome the reaction energy barrier of N_2_ activation, during the process of plasma‐catalytic NH_3_ synthesis. Thus, the translational kinetic energy of molecules, ions and free radicals in the reaction gas remains at a relatively low temperature. At the same time, the N_2_ dissociates during the plasma discharge process, prior to the adsorption process of the catalyst, and in consequence the gas‐phase substances can be adsorbed on the catalyst surface with a relatively low N_2_ binding energy. It is easy to be desorbed from the catalyst surface after the generation of NH_3_ without affecting the surface activity. Hence, the plasma‐enhanced NH_3_ synthesis can not only meet the energy requirements of the reaction at ambient temperature and pressure, allowing it to maintain thermodynamic advantage, but also break the linear relationship between binding strength of N‐containing species with catalyst surface and N_2_ dissociation [46]. Compared with the adsorption of free N_2_ or H_2_, the large amount of N and H produced during the plasma discharge greatly changes the thermal balance of NH_3_ synthesis and reaction rate. In addition, the electron bombardment and photon radiation caused by plasma discharge also accelerate NH_3_ desorption, without observable effect on the catalyst surface. In order to reveal this conclusion, Winter et al. [44]. identified the reaction intermediates adsorbed on the catalysts during the plasma‐activated NH_3_ synthesis under various temperatures using the Fourier transform infrared (FTIR) spectroscopy, as illustrated in Figure 3C. In the meantime, it is also proved that the NH_3_ yield is impacted by the plasma‐derived intermediates, together with the interaction between intermediates and catalyst surfaces, resulting in different reaction routes. Rouwenhorst et al. [45]. also confirmed that the plasma‐catalytic NH_3_ synthesis with excited N_2_ can be achieved beyond thermal equilibrium, by experimental data with ruthenium (Ru) catalysts at temperatures above 300°C. The NH_3_ yield depends on the competition between dissociative adsorption of basal N_2_ and adsorption of plasma‐generated N radical species for subsequent hydrogenation to NH_3_, along with thermocatalysis decomposition of NH_3_ (Figures 3D,E). The plasma‐catalytic NH_3_ synthesis at temperatures below 300°C is due to the adsorption of N radicals produced in the plasma, and their subsequent hydrogenation to NH_3_. These findings suggest that thermally active catalysts are not suitable for use in the plasma catalysis, because these also catalyze the reverse decomposition reactions.

## 3 Different Types of Plasma for NH_3_ Synthesis

### 3.1 Definition

It is well known that matter generally exists in three states, that is, solid, liquid, and gas. Actually, if sufficient energy continues to be added to a gas molecule, it can also be partially or completely converted into plasma state [47]. Plasma is a special state which is different with solid, liquid and gas state. It is considered to be high‐energy matter, usually containing free radicals and excited state species [48], which endow it with excellent physical and chemical properties, such as high electrical conductivity, large thermodynamic instability, and very strong coupling effect with electromagnetic field. In addition, plasma possesses high electron temperature, low gas temperature, and high energy density too. Since the concept of “plasma” was first introduced in 1928, it has been used in various fields, and fundamentally changed the development of industrial manufacture and health care [49]. Meanwhile, plasma is also regarded as one of the most promising approaches to activate the N≡N bond, because it can be carried out under ambient conditions with high productivity, thereby effectively boosting the NRR [50]. In this section, a systematic summary for recent reports on the application of different plasmas in NH_3_ synthesis process, along with their advantages and disadvantages is presented in Tables 1 and 2 respectively, in order to gain a deeper understanding of the research and development of plasma‐assisted NH_3_ synthesis technology.

**TABLE 1:** A comparison of recently reported NH_3_ synthesis processes using different plasmas.

Plasma	Catalyst	Feed gas	Power	NH_3_ yield	Energy efficiency	Ref.
			W	µmol g_cat_^−1^ min^−1^	g‐NH_3_ kWh^−1^	
DBD	0.5Ni/LaOF	N_2_:H_2_ = 1:3	13	34.94	2.7	[51]
DBD	La(OH)_3_	N_2_:H_2_ = 1:3	13	37.13	2.91	[52]
DBD	SBA‐15	Air	35	88.95	1.04	[53]
DBD	Ni‐MOF‐74	N_2_:H_2_ = 1:1	35.7	85.75	1.27	[54]
DBD	Co‐Ni/MOF‐74	N_2_:H_2_ = 1:1	34.57	43.48	0.72	[55]
DBD	Au	N_2_:H_2_ = 1:1	128.8	/	0.58	[37]
DBD	γ‐Al_2_O_3_	N_2_, H_2_O	/	0.072	0.0005	[56]
DBD	Ni/LDH‐500‐P	N_2_:H_2_ = 1:1	/	75.33	1.71	[57]
DBD	Ru/MgO	N_2_, H_2_O	/	44.5	0.065	[58]
DBD	Co‐N_2_	N_2_:H_2_ = 2:3	59	177.45	0.31	[59]
DBD	La_2_O_3_	N_2_:H_2_ = 1:3	13	35.47	/	[60]
DBD	Zeolites	N_2_:H_2_ = 4:1	6.4	2.3	1.1	[61]
GD	Pt	N_2_:H_2_ = 5:2	199l5	/	0.12	[62]
MW	/	N_2_, H_2_	1100	/	0.04	[63]
MW		N_2_, Ar, H_2_	3000	420.9	/	[64]
MW	Co/γAl_2_O_3_	N_2_, CH_4_	180	32.34	0.01	[65]
GAD	/	N_2_	/	0.67	0.026	[66]
DC	/	N_2_, H_2_O	/	5.83	0.0184	[67]

**TABLE 2:** The advantages and disadvantages of different plasma generation methods for NH_3_ synthesis.

Plasma	Advantages	Disadvantages
DBD	Atmospheric pressure, and simple equipment; Easy to integrate with catalyst; Uniform and stable discharge.	By‐product generation; High energy consumption; Carbon buildup on electrodes.
GD	High yield under low pressure; High concentration of active particles; Controllable reaction pathway.	Vacuum environment; Complex and expensive equipment; Difficult to produce continuously.
MW	No electrode contamination; High power density; Long life of active particles.	High‐cost equipment; Complex reactor; Microwave leakage.
GAD	High airflow capacity; Significant thermal effect; High energy density and strong ionization.	Extremely high energy consumption; Poor stability, and difficult to operate continuously; Severe equipment wear.
CD	Simple structure; Quick start; Atmospheric pressure.	Limited processing capacity, low efficiency and yield; Weak discharge intensity; Many by‐products.

### 3.2 Classification

Plasma can be divided into two main categories, that is, high‐temperature plasma and low‐temperature plasma. Among them, the latter can be further subdivided into thermal plasma and non‐thermal plasma. Different types of plasma exhibit significant differences in the reaction mechanisms and energy efficiency. For the high‐temperature plasma, the temperatures of electrons, ions, and background gases are all close to 10^7^ K. As for the thermal plasma, the component temperatures are uniform, and approximately 10^4^ K. Under the high‐temperature conditions of thermal plasma, the N_2_ molecules can be dissociated into active species, thereby participating in subsequent reactions. However, most of its input energy is used for gas heating rather than effective chemical reactions, resulting in a higher energy consumption compared with that of current non‐thermal plasma or catalyst‐assisted system. Furthermore, rapid quenching is required to maintain the stability of NH_3_, as high temperatures promote its decomposition. In contrast, the energy is primarily distributed to heat electrons in the non‐thermal plasma, enabling electron energies to reach about 10 eV, while the ions and background gases remain at room temperature. At this point, the gas energy is much lower than the electron energy, thereby achieving the efficient dissociation of N≡N bond under ambient conditions. Therefore, the non‐thermal plasma with low energy input is expected to significantly reduce the energy loss of conventional N_2_ fixation technology [35, 68, 69].

Furthermore, the non‐thermal plasma has the characteristics of mild conditions, simple operation, stability, safety, and low cost, so it is widely employed in activation and conversion of N_2_ [70]. Since the 1980s, scholars have conducted systematic investigations on the plasma‐assisted NH_3_ synthesis technology, involving in the reaction devices, raw materials, discharge mode and power, etc. [69]. Some of them try to increase NH_3_ yield, or reduce energy consumption by optimizing the technological parameters. In other cases, they are demonstrated that the combination of non‐thermal plasma and catalyst provides a unique condition for the transformation of N_2_ to NH_3_ [51, 71, 72, 73]. For instance, the plasma generated by reactant gas under discharge conditions can break through the equilibrium limitations of conventional NH_3_ synthesis technology, thereby producing high‐density reactants, such as ion, free radical, or vibrationally excited molecule through electron collisions, and initiating a variety of chemical reactions in the gas phase. These studies make non‐thermal plasma‐assisted NH_3_ synthesis an attractive alternative to the Haber–Bosch process. Non‐thermal plasma can be generated by the dielectric barrier discharge plasma, glow discharge plasma, microwave discharge plasma, gliding arc discharge plasma, and corona discharge plasma, etc. [51, 71, 72, 73]. Different discharge conditions and forms can produce various active species, resulting in significantly distinct activation effects.

#### 3.2.1 Dielectric Barrier Discharge Plasma

Dielectric barrier discharge (DBD) plasma requires the insertion of at least one piece of insulating medium in the air gap between two metal electrodes. The insulating medium, mainly consisted of quartz and ceramics, etc., is the key to normal discharge, which can not only limit the charge transfer during the discharge process, thereby making the discharge evenly distributed on the electrode surface, but also avoid the electrode corrosion [74]. At present, there are two modes of dielectric barrier discharge under atmospheric pressure, that is, filamentary discharge and diffuse discharge [75]. In general, the DBD plasma has relatively low ion and gas temperatures, high electron temperature, along with the generation of some high‐energy particles, thus it is able to complete discharge reactions at normal temperature and pressure. Moreover, the DBD plasma possesses features of simple device, strong operability, easy integration, and massive production, etc. [76]. Therefore, it has broad application prospects in large‐scale gas conversion, and is gradually becoming a research hotspot in the field of plasma‐assisted NH_3_ synthesis. In recent years, more and more scholars have focused their attention on the conversion of N_2_ to NH_3_ based on the non‐thermal plasma with the DBD technology as the discharge mode [77].

**FIGURE 4:**
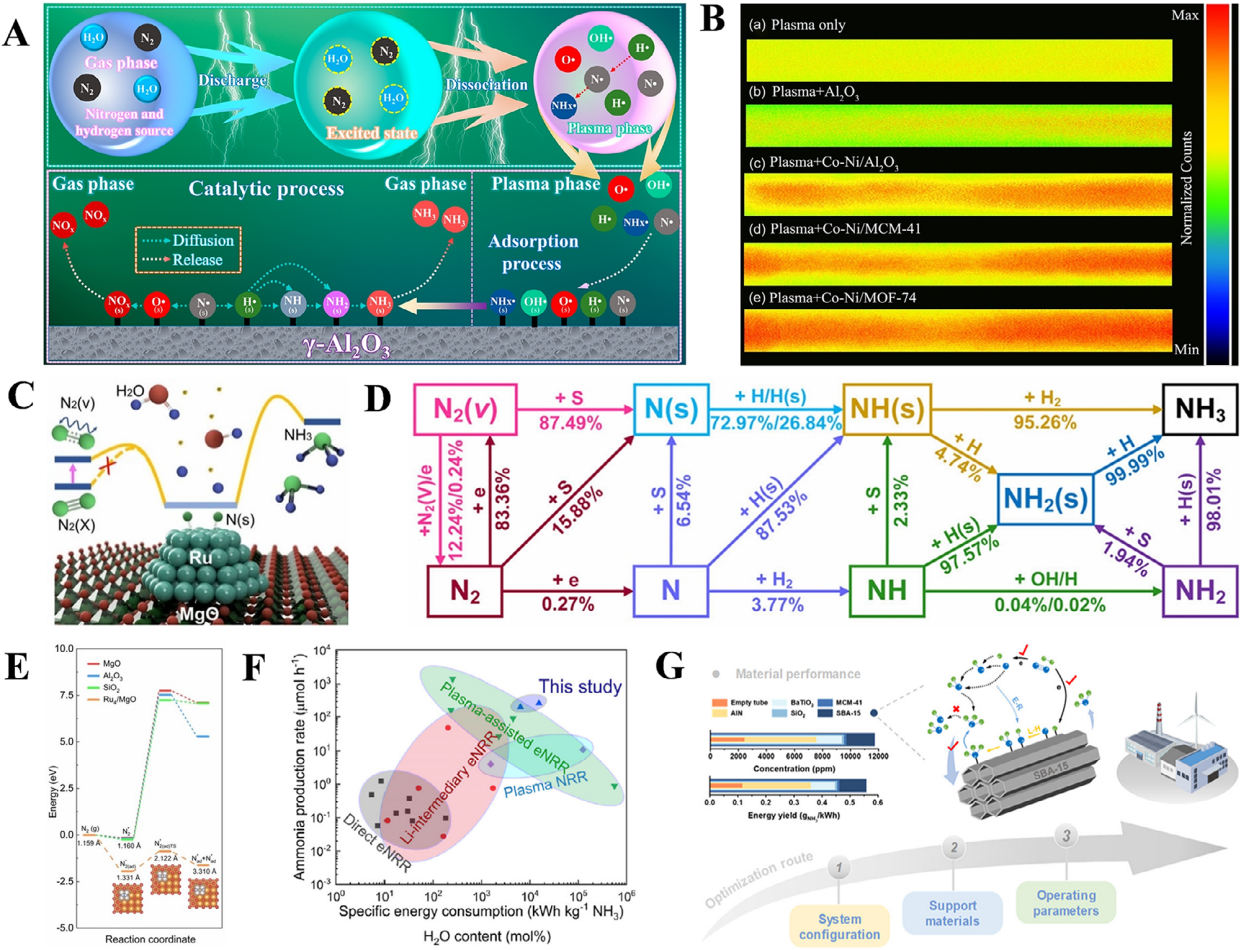
(A) Schematic illustration of reaction routes for NH_3_ synthesis by the DBD plasma combined with the γ‐Al_2_O_3_ catalyst. Reproduced with permission Ref. [56]. (B) The intensified charge‐coupled device images of plasma discharge in the absence (plasma only) and presence of different catalysts, such as Al_2_O_3_, Co‐Ni/Al_2_O_3_, Co‐Ni/MCM‐41 and Co‐Ni/MOF‐74. Reproduced with permission Ref. [55]. (C) Schematic illustration of decentralized plasma‐catalytic synthesis of NH_3_ from N_2_ and H_2_O on the Ru‐doped MgO catalyst, (D) possible reaction pathways for the plasma‐catalytic NH_3_ synthesis of N_2_/H_2_O system, (E) the N_2_ dissociation process over different catalysts, and (F) comparison of NH_3_ yield in the direct eNRR, Li‐intermediary eNRR, plasma‐assisted eNRR, plasma‐assisted NRR, and decentralized plasma‐catalytic systems. Reproduced with permission Ref. [58]. (G) Schematic diagram for the enhanced mechanism of NH_3_ synthesis on the SBA‐15 catalyst. Reproduced with permission Ref. [53].

The DBD technology can also be easily coupled with the catalyst, and show an obvious synergistic effect [78, 79, 80]. For example, the DBD can offer high‐quality energy input to the reaction system, thereby generating highly active vibrationally excited state and active specie (e.g., free radical) by ionizing gas molecules, while the catalyst may provide active sites for the reaction of active species, change the reaction path, and reduce the reaction barrier [81, 82]. In this regard, Feng et al. [56]. synthesized NH_3_ from H_2_O and N_2_ in the DBD plasma reactor filled with Al_2_O_3_ catalyst under atmospheric pressure and room temperature. The results demonstrate that the catalyst not only offers the adsorption, dissociation, and surface reaction routes towards the NRR reaction, but also significantly enhances the electric field intensity of the reactor, thereby producing more active radicals (e.g., H•, N• and NH*_x_* •) in gas phase, as illustrated in Figure 4A. Therefore, the coupling of DBD plasma and catalyst can boost NH_3_ synthesis. To match the DBD plasma reactor, Liu et al. [55]. developed a series of bi‐transition metals supported on the traditional catalysts, such as Al_2_O_3_, MCM‐41 and MOF‐74, further elevating NH_3_ yield and energy utilization. As shown in Figure 4B, the catalytic activity reflected by plasma discharge is in the order of Co‐Ni/MOF‐74 > Co‐Ni/MCM‐41 > Co‐Ni/Al_2_O_3_ > Al_2_O_3_ > plasma only. Among them, the Co‐Ni/MOF‐74 displayed the maximum yield up to 2608.70 µmol g^−1^ h^−1^ from the reaction gases of N_2_ and H_2_ with a specific energy input of 33.27 kJ L^−1^ at 200°C. Besides, the DBD plasma‐catalytic N_2_/H_2_O process on the noble metal‐based heterostructure, that is, the ruthenium cluster supported on magnesium oxide (Ru/MgO), may also an effective method for NH_3_ production under ambient conditions (Figure 4C) [58]. This is because that the high‐energy electrons can initiate the plasma catalysis through generating high‐density N_2_ (v, vapor), while ∼87.5% N_2_ (v) contributes to the generation of surface‐adsorbed N (s, solid). After that, the interaction of NH (s) and H_2_ or NH_2_ (s), together with atomic H further forms NH_3_ (Figure 4D). The catalytic activity of Ru/MgO is significantly superior to other catalysts including MgO, Al_2_O_3_, and SiO_2_, owing to the decreased activation energy towards the dissociative adsorption of N_2_ (Figure 4E), while its NH_3_ yield is even higher than those of other catalytic procedures, such as electrocatalytic NRR (eNRR), lithium (Li)‐intermediary eNRR, and plasma‐assisted NRR or eNRR (Figure 4F). Chen et al. [53]. also systematically optimized the synthetic NH_3_ ability of DBD plasma‐catalytic reaction, through adjusting the system configurations, screening catalyst support materials, and optimizing operating parameters (Figure 4G), thereby achieving a high NH_3_ yield of 5337 µmol g_cat_^−1^ h^−1^ with an energy utilization of 1.04 $g_{NH_{3}}$ kWh^−1^.

**FIGURE 5:**
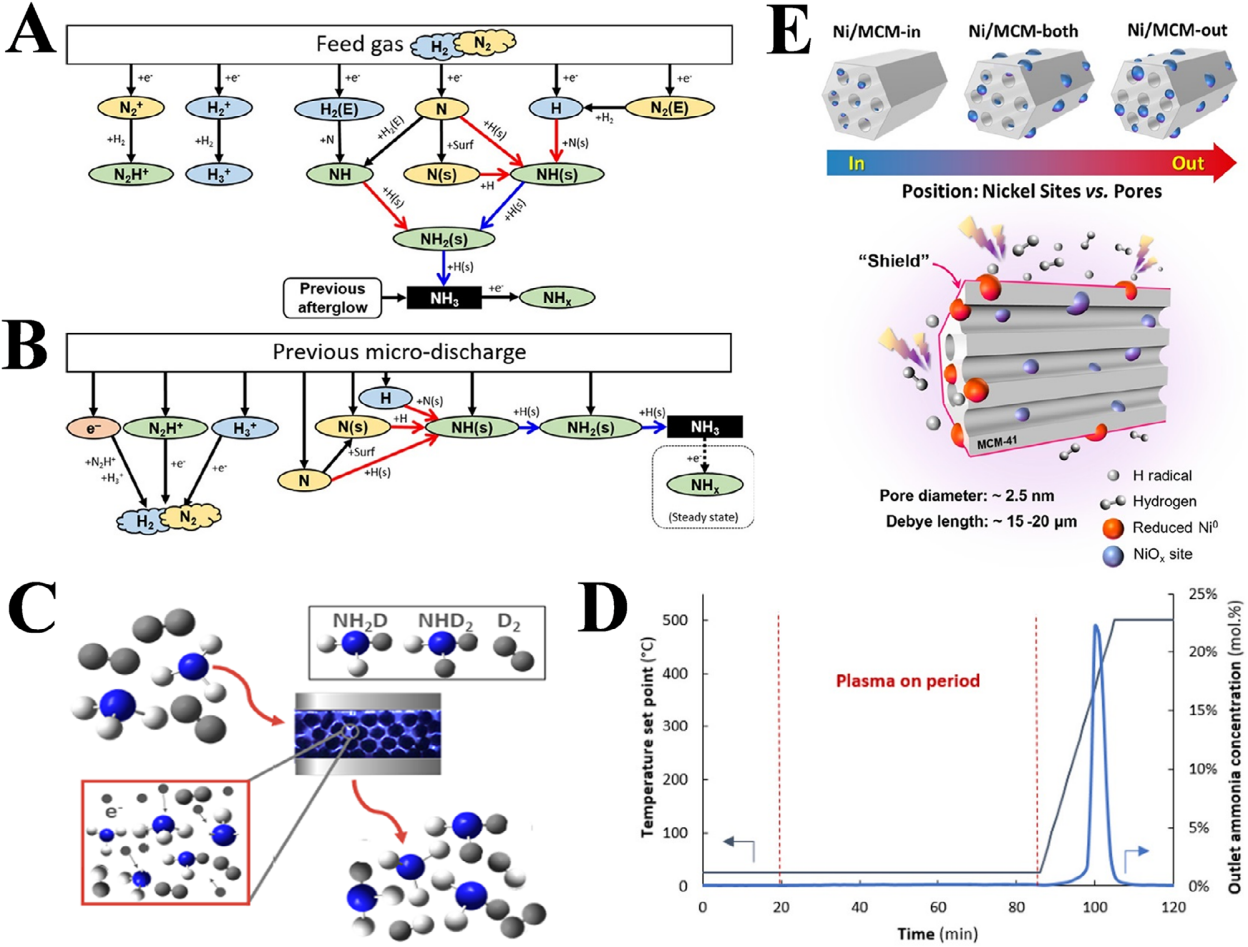
(A) Reaction mechanism of NH_3_ generation in the DBD during microdischarge, and (B) its afterglow. Reproduced with permission Ref. [83]. (C) Scheme showing the NH_3_ decomposition reaction. Reproduced with permission Ref. [84]. (D) Example experiment for plasma‐based NH_3_ synthesis with in situ adsorption. Reproduced with permission Ref. [61]. (E) Schematics of the Ni catalysts with mesoporous MCM‐41 supports (top), and shielding effect of MCM‐41 on Ni within its mesopores in the plasma discharge (bottom). Reproduced with permission Ref. [86].

Unfortunately, limitation still exists, because the energy efficiency of NH_3_ synthesis in the DBD plasma is severely limited by the decomposition of NH_3_. In this regard, van't Veer et al. [83]. employed a plasma kinetic modeling to gain insight into the formation mechanism of NH_3_ in the filled‐bed DBD reactor, and proposed that the plasma can both synthesize and decomposed NH_3_. Specifically, NH_3_ is formed from N• and H• radicals during the afterglow process, and is dissociated by electron collisions during the micro‐discharge process, as illustrated in Figures 5A,B. Further experimental evidence for the plasma decomposition of NH_3_ is also provided by Navascués et al. [84]. who reveal the deformation behavior of NH_3_ in electron collisional plasma, through the isotopic labeling experiment (Figure 5C). In view of the NH_3_ decomposition during plasma‐chemical fabrication, Peng et al. [85]. introduced a synergistic absorption strategy of magnesium chloride to decrease the influence of product deformation on the energy efficiency of the system, but its stability is poor. To this end, Kevin et al. [61]. used a relatively stable zeolite as the adsorbent for in situ recycling of NH_3_ from the DBD system, which effectively suppressed product decomposition. Correspondingly, the yield for plasma‐assisted NH_3_ synthesis with in situ recovery strategy is 2 times as large as that of steady‐state plasma‐chemical method (Figure 5D). Besides, Wang et al. [86]. also designed a Ni loaded on ordered mesoporous MCM‐41 catalyst (Ni/MCM‐41, Figure 5E), for suppressing in situ plasma‐induced NH_3_ decomposition. Owing to the absence of plasma discharges in the ordered mesopores, the generated NH_3_ could diffuse into the mesoporous MCM‐41, thus limiting the plasma‐induced reverse reaction, resulting in a high yield of more than 60 kJ L^−1^ in the DBD reactor. These encouraging works offer profound insights into the design and synthesis of the catalysts, and point out potential directions for the future development of DBD plasma‐enhanced NH_3_ synthesis.

#### 3.2.2 Glow Discharge Plasma

Glow discharge (GD) plasma is a low‐pressure discharge phenomenon, in which the working pressure is generally lower than 10 mbar. The experimental facility of GD plasma mainly consists of two parallel electrodes placed in a closed container, while its working principle is to use the electrons generated by the GD plasma to excite neutral atoms or molecules, thereby releasing energy in the form of light, as the excited particles fall from excited state to ground state. For example, Bai et al. [87]. employed an ultra‐intense narrow‐pulse electric field to form a strong GD plasma in a reactor filled with N_2_ and H_2_. Under the action of GD plasma, N_2_ and H_2_ can undergo ionization and decomposition reactions at atmospheric pressure, thus producing a large number of free atoms, ions, and free radicals, etc., and forming the non‐equilibrium plasma. Subsequently, they were finally synthesized into NH_3_ with a concentration of 5000 µL L^−1^ under the control of directional chemical reaction.

**FIGURE 6:**
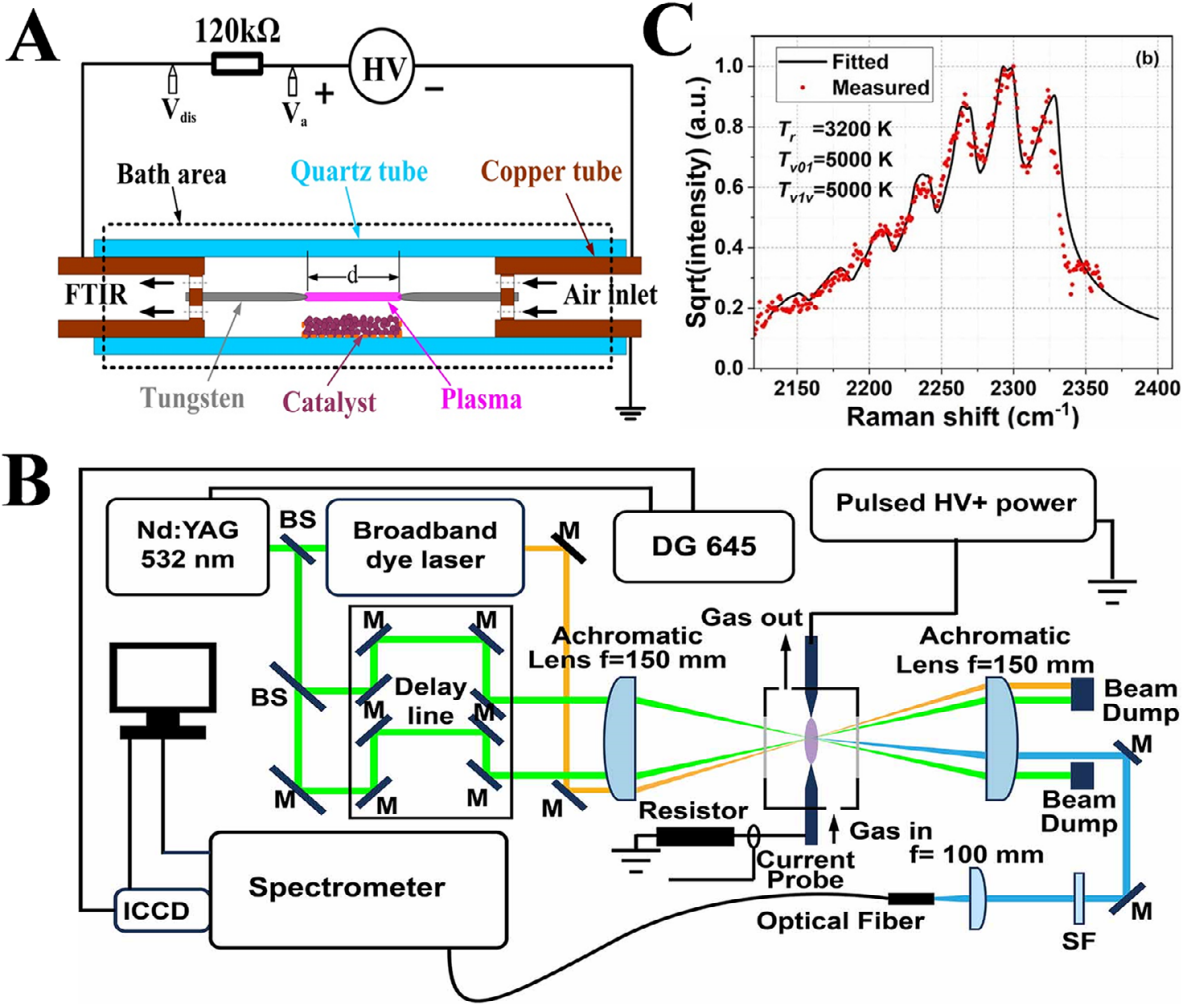
(A) Schematic illustration for the experimental apparatus of NO*_x_* production with the DC‐GD. Reproduced with permission Ref. [88]. (B) Schematic drawing for the coherent anti‐Stokes Raman scattering (CARS) test of long‐pulse air discharge, and (C) corresponding vibrational spectrum of N_2_ detected in the GD phase. Reproduced with permission Ref. [89].

In order to reduce energy cost, Pei et al. [88]. systematically investigated the effects of discharge current, gap distance, gas flowrate, tube wall temperature, and catalyst position on N_2_ fixation in atmospheric pressure GD plasma (Figure 6A), and achieved a low specific energy of 200–250 GJ tN^−1^. Previous studies have shown that vibrational excitation could reduce the energy barrier of N_2_ fixation reaction, thereby significantly improving the yield of plasma‐assisted NH_3_ synthesis. Surprisingly little has been reported on the energy efficiency of vibrational‐plasma‐excited N_2_ fixation. Qiao et al. [89]. applied vibrational coherent anti‐stokes Raman scattering as a long‐pulse air plasma to quantify the time behaviors of rotational and vibrational temperatures of N_2_ in the spark and glow modes, for overcoming the challenge of energy efficiency (Figure 6B,C). The results demonstrated that the GD can effectually heat the gas to 3500 K with a vibrational temperature exceeding 5000 K, providing important inspirations for the further investigation of plasma‐chemical N_2_ fixation derived by vibrational excitation.

Nevertheless, the limit of GD plasma‐enhanced NH_3_ synthesis still exists compared with other plasma technologies, because it generally occurs under the low pressure and rarefied gas conditions, so its discharge process is unstable. In addition, the glow can easily transition to electric arc, thereby damaging the reactor. All these disadvantages may lead to the decrease in NH_3_ yield, and therefore little has been reported on the GD plasma‐assisted NH_3_ synthesis technology.

#### 3.2.3 Microwave Discharge Plasma

**FIGURE 7:**
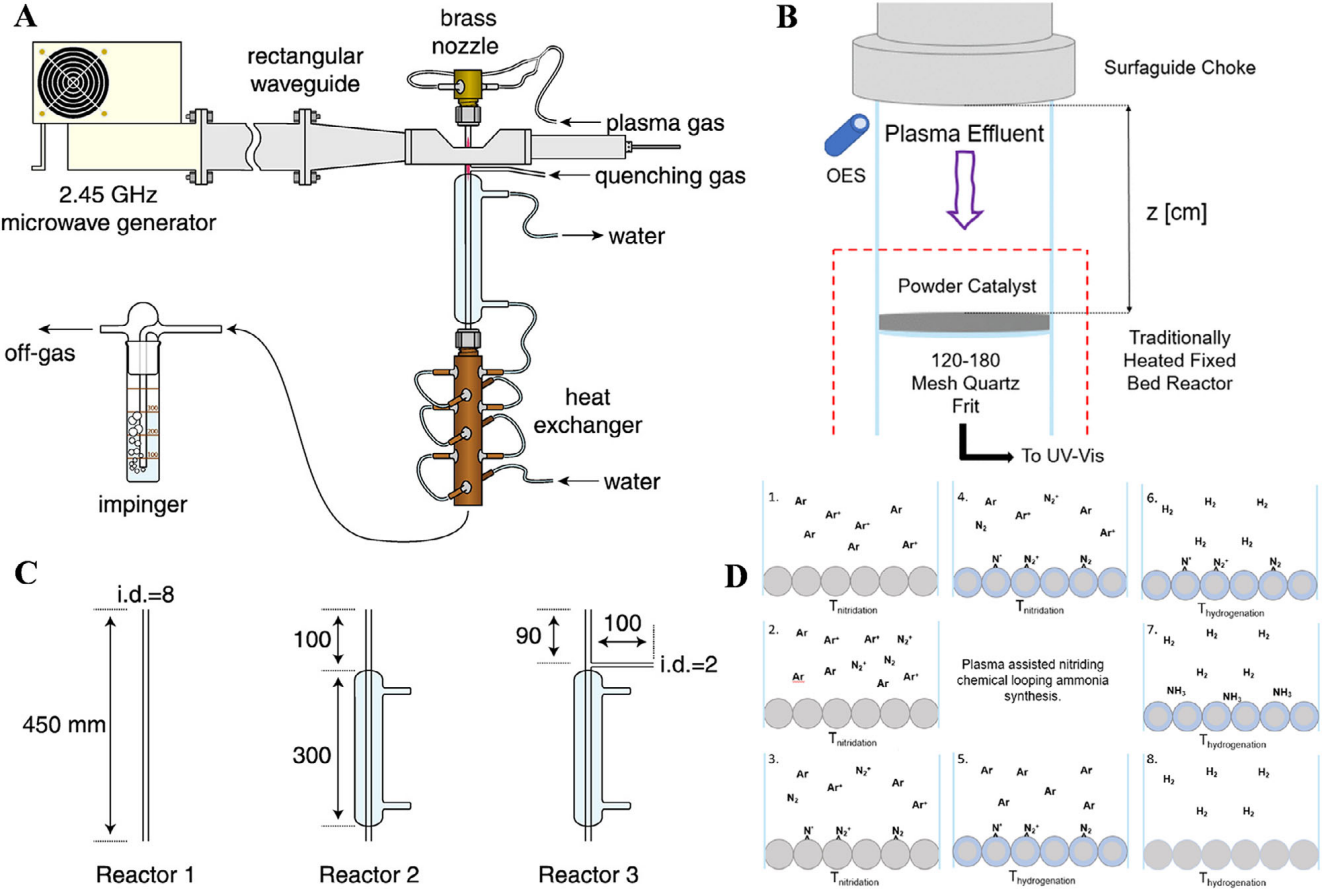
(A) Schematic illustration for the experimental apparatus, and (C) reactors of MW plasma. Reproduced with permission Ref. [63]. (B) Reactor‐catalyst‐plasma generating system, and (D) plasma‐surface mechanism. Reproduced with permission Ref. [64].

**FIGURE 8:**
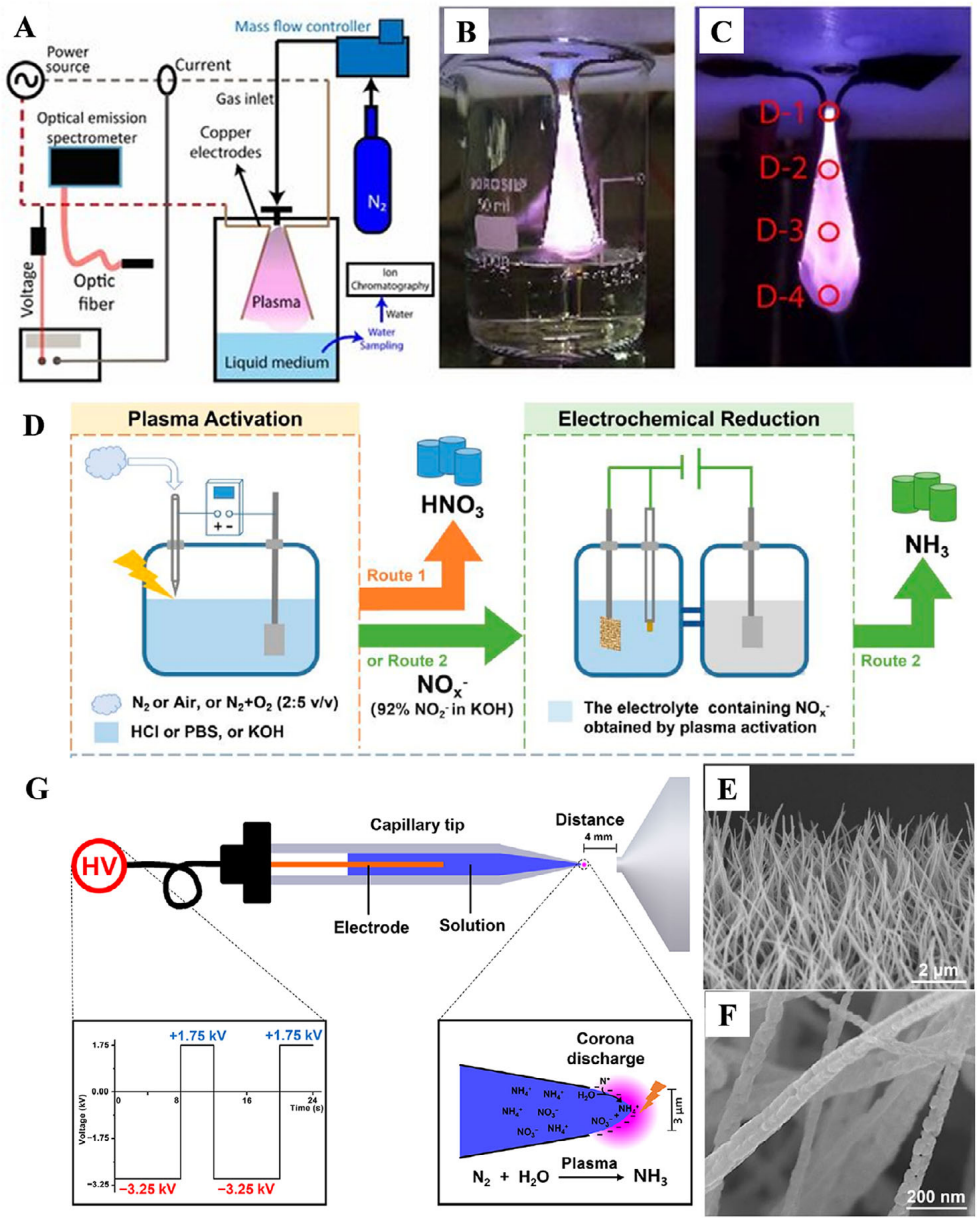
(A) Schematic illustration for the experimental setup of NH_3_ synthesis based on the GAD plasma with N_2_ as the inlet gas, (B) photograph of the GAD plasma inside the beaker interacting with H_2_O surface, and (C) snapshot of the arc captured at an exposure of 0.6 s. Reproduced with permission Ref. [66]. (D) Illustration of the integrated CD plasma and electrochemical system, and (E, F) SEM images of Cu_2_O/Cu catalyst. Reproduced with permission Ref. [96]. (G) Schematic setup of the system for NH_3_ synthesis using the PR‐nESI device. Reproduced with permission Ref. [67].

Microwave discharge (MW) plasma is produced from the gas excited by high‐frequency electromagnetic field, stemming from the standing wave resonance of microwave in resonant cavity [90]. In general, the microwave frequency is ranging from 0.3 to 10 GHz [91]. In 2008, Nakajima et al. [63]. employed atmospheric pressure microwave plasma to synthesize NH_3_, as illustrated in Figure 7A,C. The results demonstrate that the generation of active N‐containing groups is a very important factor, during the synthetic process of NH_3_. Besides, injecting H_2_ into the plasma gas may effectively inhibit the activation of N_2_. In contrast, injecting H_2_ into the downstream region of the plasma enhances the synthesis of NH_3_. Meanwhile, dilution with Ar in the plasma gas can also significantly promote the formation of N‐containing active groups. Hu et al. [64]. developed a MW plasma‐enhanced reactor (Figure 7B) to pre‐activate N_2_ before the catalytic reaction, thereby achieving the chemical cycle of NH_3_ with a high yield of 420.9 µmol min^−1^ g^−1^ under mild nitriding condition and atmospheric pressure. Figure 7D depicts the possible reaction mechanism. As shown, the MW plasma is initiated, when the catalyst bed reaches the temperature of nitridation (*T*_nitridation_, Figure 7D–1). The N_2_ is introduced into the system after the plasma is stable, followed by the nitridation reaction (Figure 7D–2). Subsequently, the activated N‐species accumulate and react with the catalyst surface (Figure 7D–3), and a nitride diffusion layer is appearance (Figure 7D–4). Once the activation processing is complete, the plasma is stopped, while the rest of N_2_ is discharged from the system with Ar flow (Figure 7D–5). Then, the system temperature is regulated to a level suitable for the hydrogenation reaction, accompanied by the introduction of H_2_ (Figure 7D–6). At the moment, NH_3_ is released from the activated species‐adsorbed surface, because of the ease of H_2_ dissociation and diffusion in bulk Fe (Figure 7D–7). At last, the NH_3_ yield gradually reduces and eventually stops, as most of the lattice N_2_ is removed (Figure 7D–8). This novel strategy based on the Fe catalyst exhibits more obviously advantages in the yield of activated species, modularity, fast startup, and low voltage input, compared with similar plasma‐catalytic technologies. The MW plasma presently still faces the challenges such as high cost and complex structure, compared to other types of plasma. Additionally, the localized micro‐discharges or Joule heating effects can easily lead to catalyst sintering. Future efforts should focus on optimizing reactor design, developing suitable catalyst, and further improving energy efficiency to promote the application of MW plasma in enhanced NH_3_ synthesis.

#### 3.2.4 Gliding Arc Discharge Plasma

The gliding arc discharge (GAD) plasma is derived from the fact that the gas is broken down under the excitation of strong electric field, thereby forming the arc at the shortest point of discharge electrode gap. Then, the arc slides along the electrode to generate a gliding arc under the action of gas flow, and periodically carries out the cyclic process of breakdown‐elongation‐arc quenching‐breakdown [92, 93]. The GAD plasma owns various features, including simple electrode structure, and easy to control, etc. In the meantime, it is also a low‐temperature plasma, which can achieve high energy density and large processing capacity and thus, the oxynitride (NO*_x_*) synthesized by the GAD plasma may obtain a higher yield than the individual plasma [94, 95]. Within a certain range of approximate field strength, up to more than 90% of electron energy in the GAD is utilized in the form of vibrational excitation. This allows most of the input energy to be used for the N_2_ activation, which is conducive to improving the reaction efficiency, and reducing energy loss. For this purpose, Indumaty et al. [66]. studied the synthesis of NH_3_ in a GAD reactor containing the N_2_ plasma and aqueous media without the catalyst, as shown in Figure 8A. During the GAD process, a plasma plume was formed, and it could be observed vibrational and rotational excitations on the top of plasma plume in contact with water below (Figure 8B), thereby boosting the NH_3_ formation. The NH_3_ concentration was 2.12–5.69 ppm, while its production rate varied in the range of 0.63–0.68 mg h^−1^, depending on the plasma exposure time. This is confirmed by an increase in the vibrational temperature from 2632 K (Figure 8C, D‐1 zone) to 3778 K (Figure 8C, D‐4 zone), meaning more enhanced electron energy distribution of vibrational excitation (∼24%), compared with the electronic excitation (∼0.03%). The maximum productivity of this GAD reactor is 0.68 mg h^−1^ with an energy efficiency of 26.8 mg kWh^−1^. This work therefore demonstrates that the GAD plasma can produce green NH_3_ in the absence of catalyst. The GAD plasma requires a strong electric field to form and maintain an arc for now, which slides along the electrode surface. This leads to the issues, for example, high energy consumption, easy damage to the catalyst surface, difficulty in continuous operation, and severe wear of equipment. Therefore, it is usually not suitable for NH_3_ synthesis using N_2_, H_2_ or H_2_O as raw materials.

#### 3.2.5 Corona Discharge Plasma

Corona discharge (CD) plasma is a locally self‐sustained discharge phenomenon, occurring in the uneven electric field formed by the gas medium at asymmetric electrodes under atmospheric or high‐pressure conditions. When a voltage slightly lower than the breakdown voltage is added to two discharge electrodes, while the local field strength near the electrode surfaces also exceeds the ionizing field strength of the gas, the gas medium near the electrodes is ionized or dissociated by the localized breakdown. At the same time, the gas insulation is destroyed, and the internal resistance decreases, together with a dim glow appearance around the electrode, that is the CD phenomenon [97, 98]. In this respect, Luo et al. [96]. reported a plasma electrolysis system, in which the plasma was generated by an air‐pulsed positive direct current CD (Figure 8D), and selectively synthesized either HNO_3_ or NH_3_ over the Cu_2_O nanowire electrocatalyst (Figure 8E,F) from the air through an electrolyte conditioning strategy. The lowest total energy consumption for the synthesis of NH_3_ is 3.91 kWh mol^−1^. Fang et al. [67]. also proposed the in‐situ synthesis and measurement of NH_3_ from N_2_ by the nanoelectrospray‐based CD plasma coupled with the mass spectrometry. The reaction and analysis are achieved via a polarity reversal nanoelectrospray ionization (PR‐nESI) device, as presented in Figure 8G. For instance, the plasma is generated by the CD at the capillary tip during the negative polarity half‐week, and then N_2_ is immobilized through the interfacial reactions and converted to NH_3_ in liquid H_2_O. It is found that the distance between plasma and H_2_O surface plays a decisive role in N_2_ immobilization, due to the short lifetime of active plasma. This study was carried out in the presence of plasma with both inverted polarity and plasma, in which the plasma was generated on the surface of a Taylor cone, and the plasma was in direct contact with the H_2_O surface. The whole process ensured effective N_2_ fixation and NH_3_ synthesis. The CD plasma has become an effective tool for exploring the mechanisms of plasma‐catalytic NH_3_ synthesis, because of its simple setup structure and inexpensiveness. However, its shortcomings, such as weak discharge intensity, low energy efficiency, poor stability, and by‐product generation, result in a weaker competitiveness compared with those of other plasma technologies.

## 4 Synergistic Effect of Plasma and Catalysis

### 4.1 Plasma Coupled With Electrocatalysis

**FIGURE 9:**
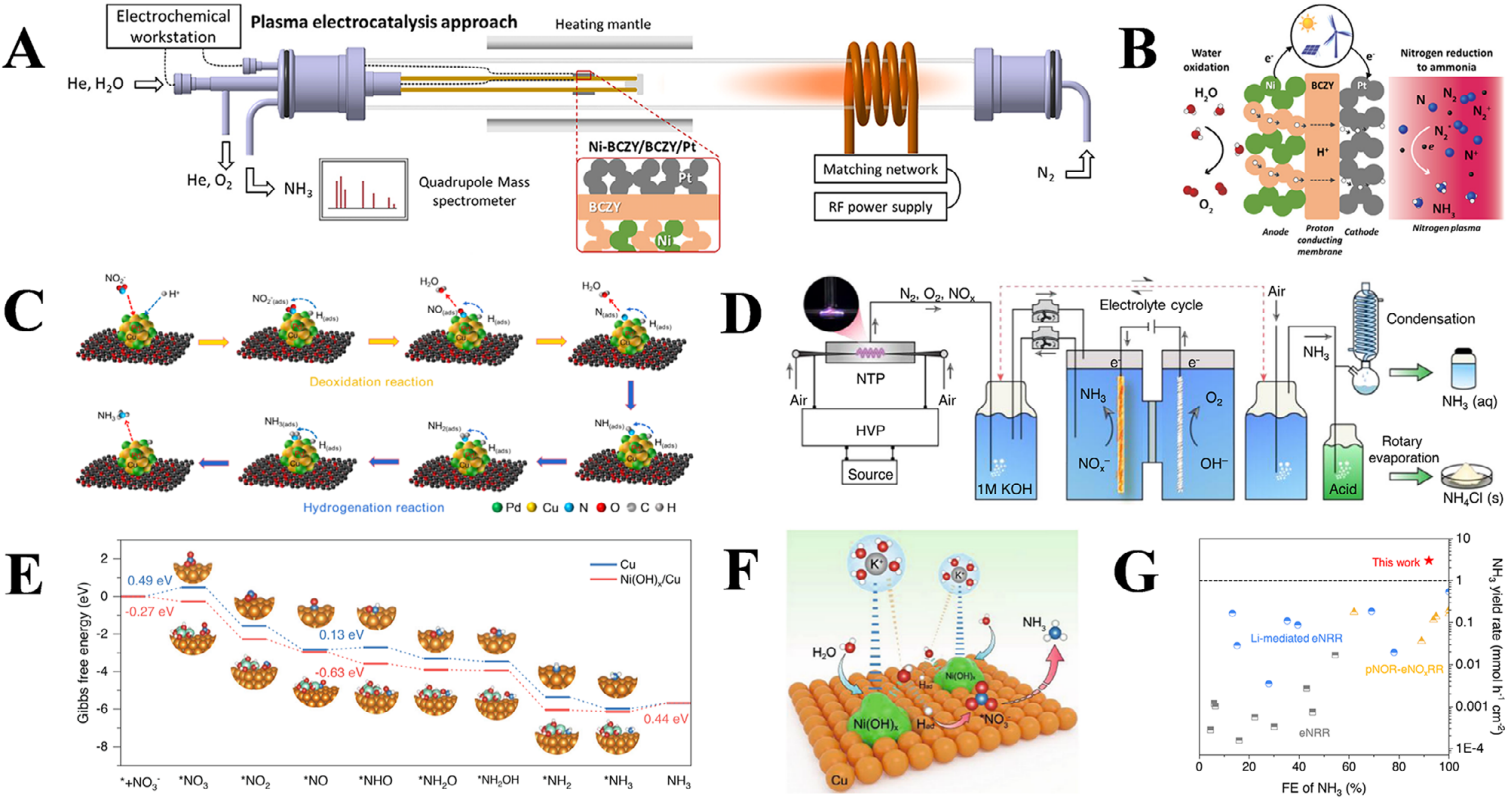
(A) Schematic representation of hybrid plasma electrochemical reactor, and (B) NH_3_ production from N_2_ and H_2_O using a plasma‐activated proton conducting solid oxide electrolyzer. Reproduced with permission Ref. [99]. (C) Schematic illustration of the eNO_2_^−^RR mechanism on the Cu_2_Pd/CBC catalyst. Reproduced with permission Ref. [100]. (D) Schematic of pNOR‐eNO*_x_* ^−^RR system and NH_3_ products collection, (E) Gibbs free energy diagram of eNO_3_^−^RR on the Cu and Ni(OH)*_x_*/Cu catalysts, (F) schematic illustration for the role of Ni(OH)*_x_* species in promoting H_2_O activation and N‐containing intermediates hydrogenation on Cu, and (G) comparison of the NH_3_ yield and FE with previous reports, including the eNRR, Li‐mediated eNRR, and pNOR‐eNO*_x_* ^−^RR. Reproduced with permission Ref. [101].

Recently, the plasma‐integrated electrocatalysis is recognized as an alternative system to the traditional electrocatalytic process towards the NH_3_ synthesis from N_2_ and H_2_O. In this system, the plasma is used for activating N_2_, thereby reducing the energy barrier of subsequent electrocatalytic NRR. For this purpose, Sharma et al. [99]. established a plasma‐assisted electrochemical device (Figure 9A), in which the H_2_ produced from the oxidation reaction of H_2_O on anode is transported to cathode via a proton conducting membrane (Figure 9B), and subsequently reacts with the plasma‐activated N_2_ to form NH_3_ with yield and Faradaic efficiency (FE) of up to 26.8 nmol s^−1^ cm^−2^ and 88%, respectively. Li et al. [100]. also proposed an integrated strategy combining low‐temperature plasma technology with electrochemical reduction, in which the plasma firstly drove the air activation to form nitrite ion (NO_2_^−^), followed by an electrochemical NO_2_^−^ reduction reaction (NO_2_^−^RR) to produce NH_3_ with a yield of 1956.65 µg h^−1^ mg^−1^. The possible NO_2_^−^RR route is also proposed, wherein the reaction involves in three steps of deoxidation and hydrogenation reactions, as illustrated in Figure 9C. Similarly, Liu et al. [101]. also proposed a tandem system of plasma‐driven N_2_ oxidation reaction coupled with electrochemical NO*_x_* reduction reaction (pNOR‐eNO*_x_* ^−^RR, Figure 9D), and revealed that the adsorption and hydrogenation processes of reactive nitrogen species (NO*_x_^−^*) occurred more easily on the Ni(OH)*_x_*/Cu than on the Cu surface (Figure 9E). It is because the Ni(OH)*_x_* on Cu enriches hydrated cations (K^+^) in the double layer, owing to the noncovalent interactions. And the H_2_O near the catalyst surface can be facilely adsorbed and dissociated on their interfaces, thereby producing H_ad_ to take part in the subsequent hydrogenation reactions of N‐containing intermediates adsorbed on the Cu towards NH_3_ (Figure 9F). Therefore, the pNOR‐eNO*_x_* ^−^RR system based on the Ni(OH)*_x_*/Cu catalyst may achieve a high NH_3_ yield of 3 mmol h^−1^ cm^−2^ along with a FE of 92%, which outperforms those of previously reported Li‐mediated eNRR and eNRR processes (Figure 9G).

Moreover, Meng et al. [102]. synthesized the nitrates/nitrites (NO*_x_* ^−^), through introducing a titanium (Ti) bubbler into the air plasma activation system, and directly used the obtained NO*_x_* ^−^ aqueous solution as a cathodic electrolyte for the following electrocatalytic NO*_x_* ^−^RR over the cobalt oxide (Co_3_O_4_) nanoparticles enriched with oxygen vacancies. This novel plasma‐electrocatalytic system exhibited remarkable NH_3_ yield of 39.60 mg h^−1^ cm^−2^ and corresponding high FE of 96.08%. Liu et al. [103]. also developed a pNOR‐eNO*_x_* ^−^RR system based on the highly dispersed copper‐iron (Cu‐Fe) nanoalloy for directly synthesizing NH_3_ from air, which exhibited high NH_3_ yield (190.46 µmol h^−1^ cm^−2^) and FE (93.74%).

In general, the high energy consumption of plasma‐assisted catalysis is considered to be the keypoint where the improvement is the most needed. Now, it has been well demonstrated that the energy efficiency of N_2_ activation could be significantly improved by combining plasma with electrochemical process, thereby reducing the energy barrier for subsequent NRR. This is mainly due to the fact that the plasma‐electrocatalytic integrated strategy can achieve the leap in energy efficiency of NH_3_ synthesis, through the efficient utilization of vibrationally excited N_2_. At the same time, the new interfacial reaction mechanism also releases its application potential. In addition, its distributed system powered by renewable energy can further reduce the cost of green NH_3_, approaching the critical point of the coal‐based H‐B process. Hence, the hybrid system coupling plasma and electrochemical process may offer an opportunity to reduce energy consumption, and achieve satisfactory results in terms of NH_3_ yield and FE [104]. Although the plasma coupled with electrocatalytic technology shows the potential to break through traditional energy barriers, its large‐scale application still faces many practical challenges and path dependencies. For instance, the non‐uniform transfer of plasma‐generated active nitrogen species at the gas–liquid–solid phase interface results in insufficient utilization of reaction sites on the electrode surface, which remains the primary bottleneck of interfacial mass and energy transfer in the plasma‐electrocatalytic system. The high price of noble metal catalysts may restrict its industrial applications, as well. Furthermore, energy matching and process intensification are issues that need to be considered in the distributed NH_3_ production industry. It is believed that the industrialization process of plasma‐electrocatalytic NH_3_ synthesis will eventually become a reality, with the maturity of technologies, such as the optimization algorithm of pulse discharge time and sequence, anti‐plasma corrosion electrode as well as coating, etc.

### 4.2 Plasma Coupled With Photocatalysis

**FIGURE 10:**
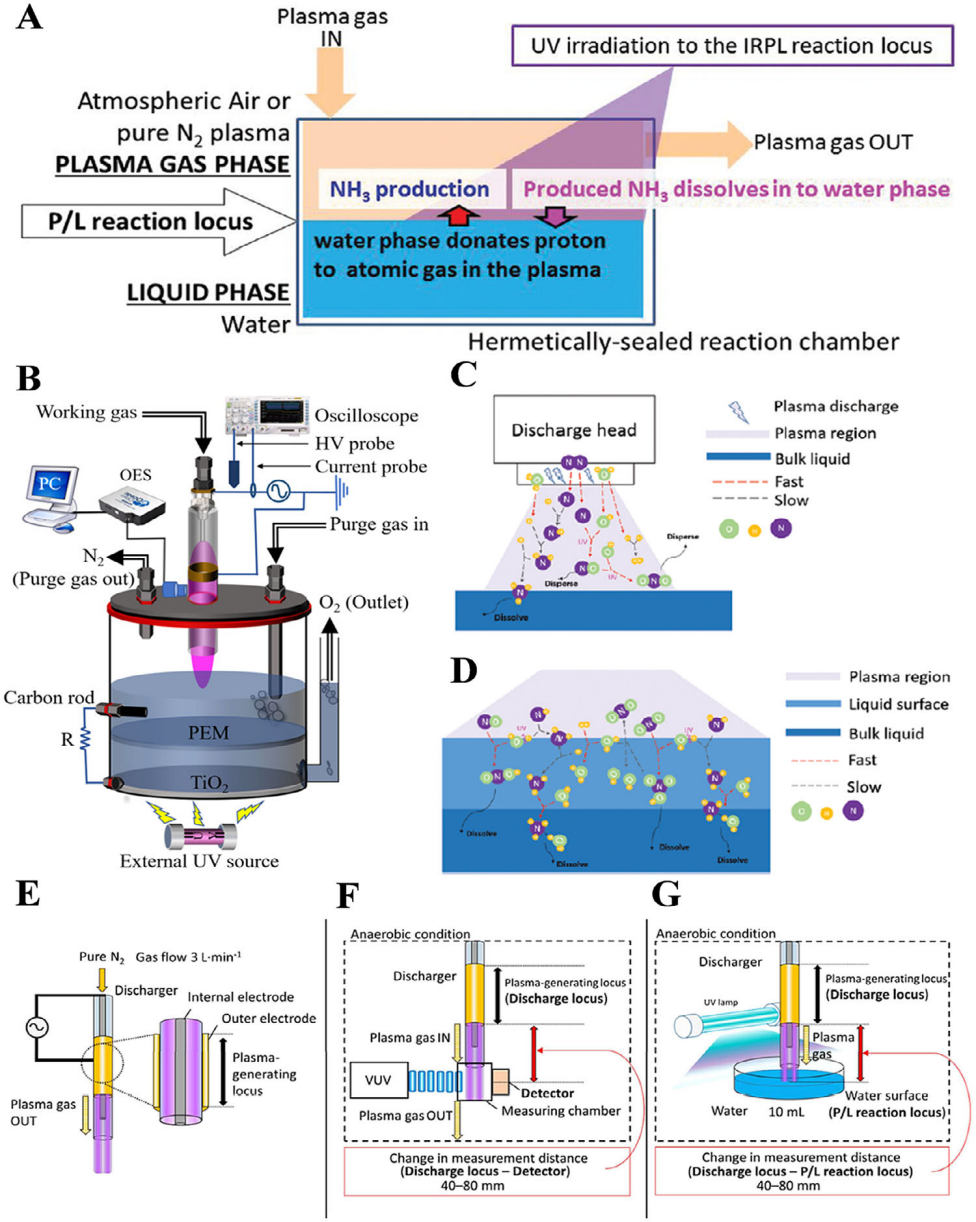
(A) Construction of the interfacial reactor between plasma phase and liquid phase (P/L) for NH_3_ synthesis. Reproduced with permission Ref. [105]. (B) Schematic diagram of N_2_ plasma jet impinging on the electrolyte at the cathode side of photo‐electrochemical H_2_O splitting reaction cell together with a proton exchange membrane (PEM). Reproduced with permission Ref. [106]. (C) Mechanism for the in situ jet plasma N_2_ fixation in the gas phase, and (D) at the liquid surface. Reproduced with permission Ref. [107]. (E) Illustration of dielectric barrier discharge device for the excited N_2_ gas production, and (F) pure N_2_ is introduced into the discharger to produce N‐plasma, which is introduced into the measuring chamber, or (G) H_2_O in a flat glass dish. Reproduced with permission Ref. [108].

In 2016, Haruyama et al. [105]. proposed a new NH_3_ synthesis approach. The biggest difference from the above methods is that the reaction occurs at the surface of plasma gas phase and H_2_O liquid phase under the irradiation of ultraviolet (UV) light. The H_2_ source is from H_2_O during the reaction to synthesize NH_3_. Besides, the authors innovatively introduced the photo‐chemical reactions into the plasma‐enhanced NH_3_ synthesis process under ambient temperature and pressure without any catalyst (Figure 10A). For instance, the ionized plasma N_2_ (gas phase) is passed into a reactor containing H_2_O (liquid phase), and then the plasma‐liquid (P/L) interface is irradiated with the UV light at wavelengths of 185 nm and 254 nm to synthesize NH_3_. During this P/L reaction, the synthetic pathway may mainly involve in the following three steps: (i) the N_2_ in the plasma atmosphere first generates a large number of activated state atomic nitrogen (N) through the inelastic collisions; (ii) subsequently, the plasma‐activated N is in contact with the liquid‐phase H_2_O to form NH• under the UV irradiation, followed by NH_2_• formation at the P/L interface; (iii) finally, the NH_2_• further reacts with H_2_O to produce NH_3_. Lamichhane et al. [106]. further combined photo‐electrochemical H_2_O splitting with plasma system, wherein the N generated by the plasma may react electrochemically with hydrogen ions (H^+^) obtained from the UV‐induced H_2_O decomposition, thereby producing NH_3_ on the P/L interface, as depicted in Figure 10B. This synergistic strategy can be operated under ambient temperature and pressure using the N_2_ and H_2_O as the raw materials without any catalyst or sacrificial reagent, while the NH_3_ yield is about six times as large as that of individual plasma. Furthermore, Peng et al. [107]. used the spray‐type jet plasma and UV light to synergistically excite vapor and liquid H_2_O molecules for boosting N_2_ fixation, which significantly raised the yields of nitrite (NO_2_^−^), nitrate (NO_3_^−^) and NH_3_ with the highest values of 15.1, 40.3 as well as 2.5 mmol min^−1^, respectively. The co‐synthesis of NO_2_^−^, NO_3_^−^ and NH_3_ is probably ascribed to the gas phase reaction, liquid phase reaction, as well as their interaction. Specifically, in the gas phase reaction, the electron excites N_2_ and vaporized H_2_O to produce nitrogen free radicals and hydroxyl radicals, respectively (Figure 10C). While in the liquid phase reaction, the N_2_ along with its related free radicals reacted with H_2_O to further generate NH_3_ under the irradiation of UV light (Figure 10D).

In addition to the N, there are many reactive nitrogen species existing in the plasma, such as excited state N_2_^*^ and nitrogen molecular ions (N_2_^+^) classified according to excitation energy level, and they have different lifetimes and reactivity. Sakakura et al. [108]. quantified the discharge‐excited N, N_2_^*^ as well as N_2_^+^, and demonstrated experimentally the effect of reactive nitrogen species with different lifetime, stability and property, along with their plasma‐enhanced photo‐catalytic reaction mechanism, separately. Figure 10E–G schematically illustrated the experimental conditions of vacuum ultraviolet/ultraviolet (VUV/UV) radiated P/L interfacial reaction. Among them, the plasma is obtained from the DBD (Figure 10E), and the stability of N is measured through changing the distance between discharge locus and photo‐detector (Figure 10F), while the relationship between NH_3_ yield and P/L reactor is also investigated by varying the distance between discharge locus and H_2_O surface (Figure 10G). When the VUV/UV radiation was employed during the reaction between plasma gas phase and aqueous phase, the UV irradiation could promote the N‐compounds formation from N_2_^*^ and N_2_^+^ instead of N. These results suggest that the P/L interfacial reactions starting from N or N_2_^*^ and N_2_^+^ follow different reaction mechanisms, and the UV‐irradiated aqueous phase may generate H• and •OH, thereby hastening NH_3_ synthesis.

### 4.3 Plasma Coupled With Thermocatalysis

**FIGURE 11:**
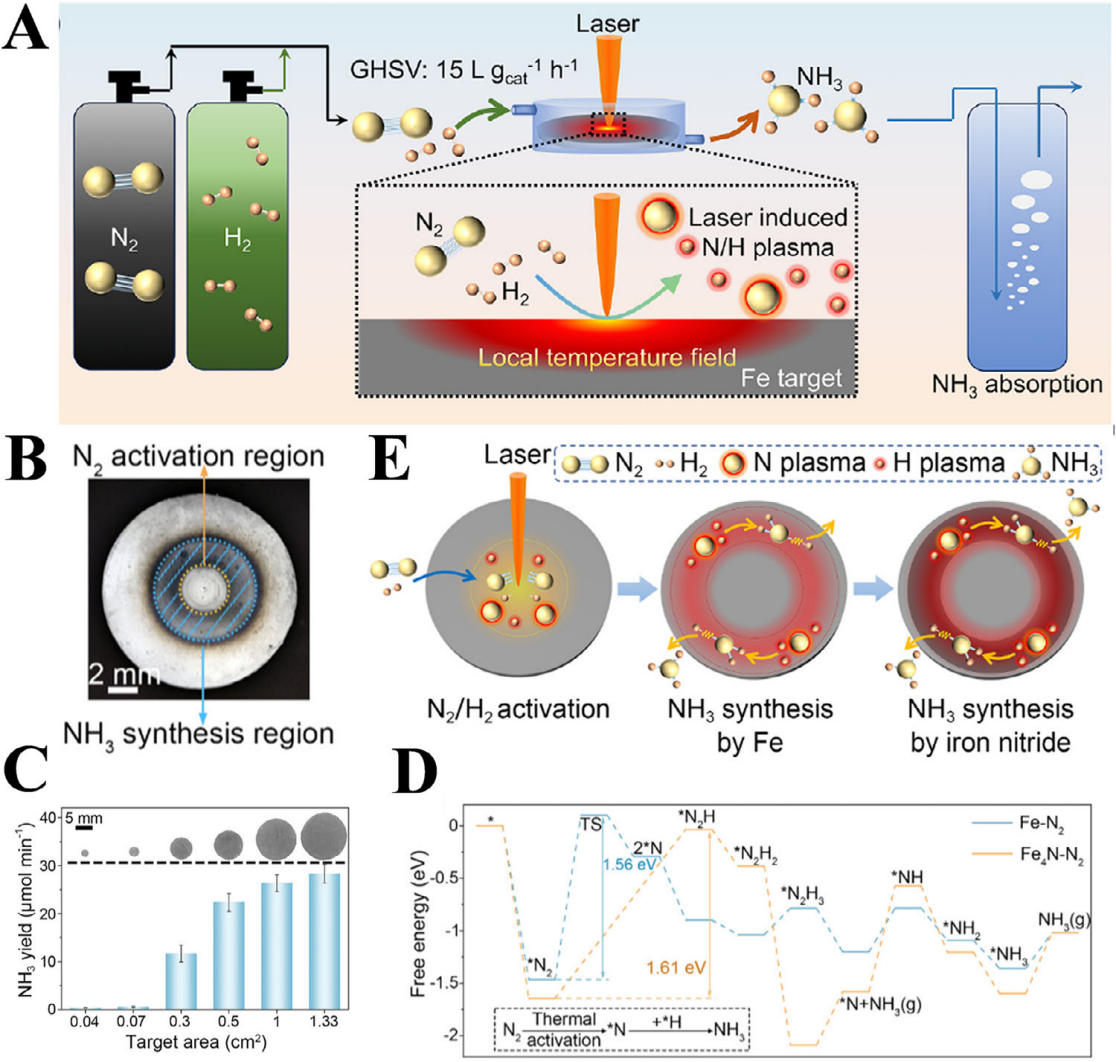
(A) Schematic exhibition of the LIAS process, (B) the optical image of different functional regions on the Fe target, (C) the NH_3_ yield with different Fe acreage, (D) the energy profiles of NH_3_ synthesis process based on the DFT calculations, and (E) schematic diagram of catalytic mechanism of the LIAS process. Reproduced with permission Ref. [110].

Although the N_2_ activation process under mild conditions favors the thermodynamics of NH_3_ synthesis, a proper temperature also plays a key role in accelerating its kinetics. In this respect, Jensen et al. [109]. elevated the efficiency of plasma‐assisted NH_3_ synthesis by 20% through raising the operating temperature from 100°C to 200°C, proving that appropriate temperature increase could significantly promote the synthesis rate of NH_3_. Wu et al. [110]. also presented a novel NH_3_ synthesis system combined laser‐induced plasma and laser‐generated local temperature field, which was significantly superior to that of conventional thermocatalysis. Figure 11A schematically illustrates this laser‐induced ammonia synthesis (LIAS) device, in which a high‐temperature center is generated at the focal point of target under the laser irradiation, as shown in Figure 11B. The surrounding gas can be collided and ionized into plasma via the free electrons excited by the target, and meanwhile the laser irradiation creates a localized temperature field and a gradient temperature field through thermal diffusion, which meet the requirements of N_2_ activation and NH_3_ synthesis at different temperatures. And the NH_3_ yield of LIAS process can be increased with the acreage of central region (Figure 11C). The LIAS achieves an initial NH_3_ yield of 70.8 µmol g^−1^ min^−1^ accompanied by a high H_2_ conversion of 9.5% with Fe as catalyst, and a stable yield of 23.63 µmol g^−1^ min^−1^ with a H_2_ converting ratio of 3.2% using iron nitride (Fe_4_N), because the energy barrier of rate‐limiting step on Fe is smaller than that on Fe_4_N (Figure 11D). As for their reaction mechanism, the N_2_ is ionized into N plasma in the central region by laser radiation over Fe, while the N plasma is migrated to the annular region for NH_3_ synthesis under the catalytic effect of Fe and Fe_4_N (Figure 11E).

Recently, Hu et al. [111]. designed an environmentally friendly plasma thermocatalysis process composed by the DBD reactor and the M/CeO_2_ or M/MgO (M = Ru, Fe) catalyst, for sustainable synthesis of NH_3_ at atmospheric pressure. The highest NH_3_ yield of 6.8 mmol g^−1^ h^−1^ is achieved at 400°C on the Ru/CeO_2_, which is far superior to those reported in the literatures using the DBD reactor alone. In addition to the plasma‐mediated gas‐phase and catalytic surface reactions, the increase in NH_3_ concentration under the plasma‐catalyzed conditions may be attributed to the synergistic effect between plasma and catalyst, wherein the plasma promotes the desorption of NH_3_ and NH*_x_*. Muzammil et al. [112]. also developed a hybrid system combined plasma‐generated NO*_x_* with thermocatalysis process, which delivered higher NH_3_ selectivity (95%) and yield (7348 mg h^−1^) than those of other plasma‐catalytic approaches. Table 3 summarizes the development status of electrocatalysis, photocatalysis, and thermocatalysis coupled with plasma system for NH_3_ synthesis in detail. These studies demonstrate that the performance of NH_3_ synthesis can be effectively enhanced through the precise control of temperature field, synergistic effect of plasma and thermal catalysis, and innovative reaction pathway. In the meantime, they also offer insights into the design of efficient reactors and matching catalysts that integrate the temperature control, plasma activation, and optimization of catalytic reaction, thereby pointing the way to future development of efficient and sustainable NH_3_ synthesis technologies.

**TABLE 3:** Electrocatalysis, photocatalysis, or thermocatalysis coupling with plasma system for NH_3_ synthesis.

Type	Catalyst	Electrolyte	NH_3_ yield	FE (%)	Condition	Ref.
Electrocatalysis	Au/TiO_2_	0.1 m HCl	1258.8 µmol g_cat_^−1^ h^−1^	8.11	−0.2 V versus RHE	[113]
	TiO_2_/rGO	0.1 m Na_2_SO_4_	890 µmol g_cat_^−1^ h^−1^	3.3	−0.9 V versus RHE	[114]
	Ru SAs/N‐C	0.05 m Na_2_SO_4_	7111.8 µmol g_cat_^−1^ h^−1^	29.6	−0.3 V versus RHE	[115]
	Cr_2_O_3_/CP	0.1 m Na_2_SO_4_	1488.2 µmol g_cat_^−1^ h^−1^	6.78	−0.9 V versus RHE	[116]
	B_4_C/CPE	0.5 m H_2_SO_4_	1562.9 µmol g_cat_^−1^ h^−1^	15.95	−0.75 V versus RHE	[117]
Photocatalysis	CuCr‐LDH	Water	73.5 µmol g_cat_^−1^ h^−1^	/	Full spectrum	[118]
	g‐C_3_N_4_‐V_N_	Methanol	1240 µmol g_cat_^−1^ h^−1^	/	*λ* >420 nm	[119]
	Bi_5_O_7_Br	Water	1380 µmol g_cat_^−1^ h^−1^	/	*λ* > 400 nm	[120]
	BiOCl	Methanol	92.4 µmol g_cat_^−1^ h^−1^	/	Full spectrum	[121]
	MoS_2_	Water	325 µmol g_cat_^−1^ h^−1^	/	*λ* > 420 nm	[122]
Thermocatalysis	Co/BaCe_3−_ *_x_* N*_y_* H*_z_*	/	10100 µmol g_cat_^−1^ h^−1^	/	673 K	[123]
	Ru/Ca(NH_2_)_2_	/	15800 µmol g_cat_^−1^ h^−1^	/	503 K	[124]
	Mn‐LiH	/	11800 µmol g_cat_^−1^ h^−1^	/	650 K	[125]
	Co‐LiH	/	4600 µmol g_cat_^−1^ h^−1^	/	573 K	[126]
	Ru/BaTiO_3_	/	19360 µmol g_cat_^−1^ h^−1^	/	654 K	[127]
Plasma + electrocatalysis	Cu_10_Fe_1_‐CFP	0.5 m Na_2_SO_4_	190.46 µmol cm_cat_^−2^ h^−1^	93.74	−0.53 V versus RHE	[103]
	Co_3_O_4_	1.0 m NaOH	2329.4 µmol cm_cat_^−2^ h^−1^	96.08	−0.8 V versus RHE	[102]
	N‐MoS_2_/VGs	0.1 m KOH	429.4 µmol cm_cat_^−2^ h^−1^	38.1	−0.53 V versus RHE	[128]
	Co SAs/N‐C	0.1 m KOH	84.1 µmol cm_cat_^−2^ h^−1^	100	−0.33 V versus RHE	[129]
	Cu_2_Pd/CBC	0.5 m KOH	115.1 µmol mg_cat_^−1^ h^−1^	93.79	−0.2 V versus RHE	[100]
	PtCu NP	1.5 m KOH	9.8 µmol mg_cat_^−1^ h^−1^	84.37	0 V versus RHE	[130]
Plasma + photocatalysis	/	Water	2.5 µmol min^−1^	/	*λ* = 254 nm	[107]
	/	Water	1.17 µmol min^−1^	/	/	[105]
	TiO_2_	0.01 m H_2_SO_4_	/	/	*λ* = 319 nm	[106]
Plasma + thermocatalysis	Fe	/	4248 µmol g_cat_^−1^ h^−1^	/	/	[110]

### 4.4 Synergistic Effect Between Plasma and Catalyst

**FIGURE 12:**
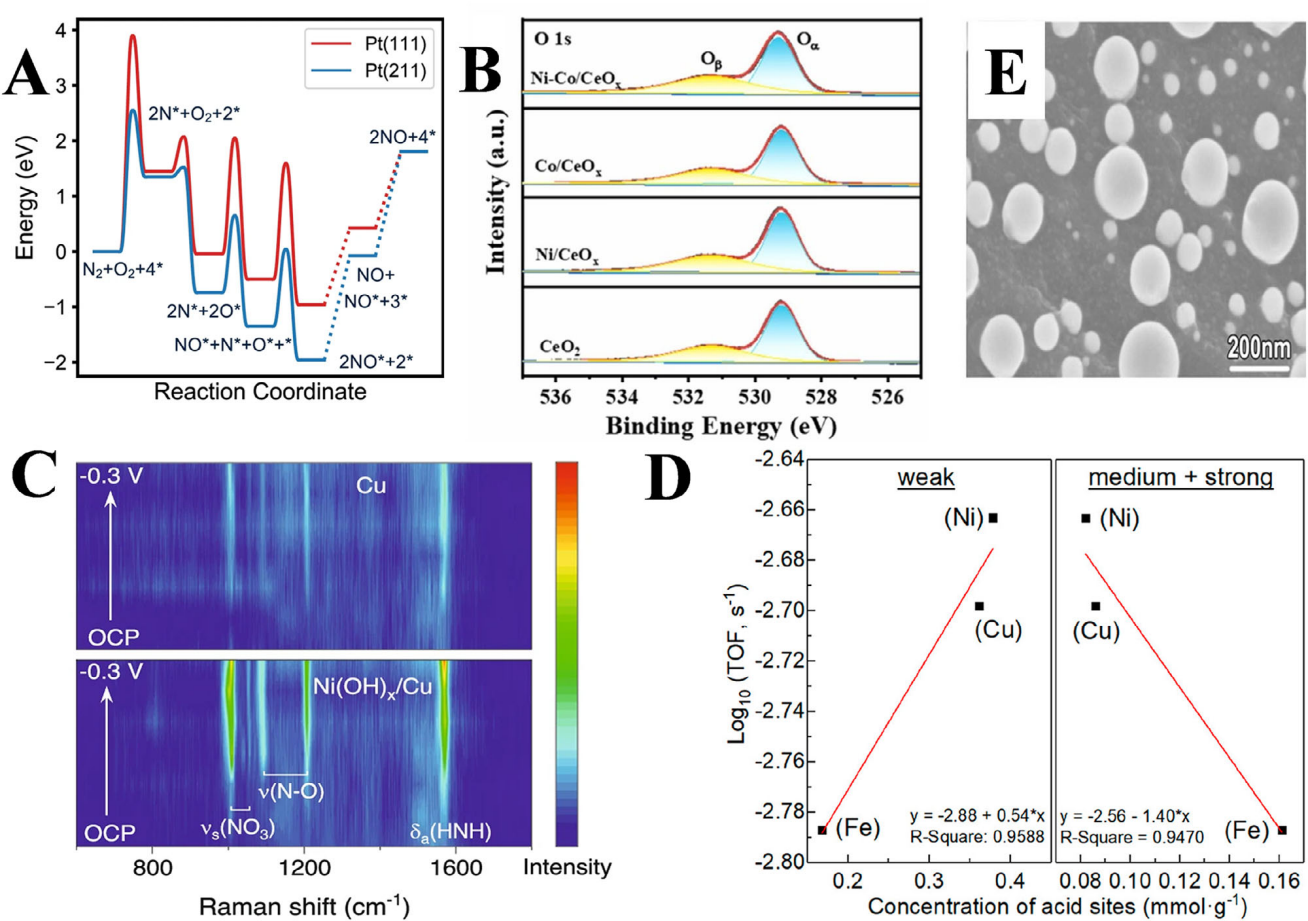
(A) The DFT‐reported potential energy surfaces for thermal N_2_ oxidation on Pt (211) and Pt (111). Reproduced with permission Ref. [131]. (B) The high‐resolution O 1 s spectra of CeO_2_, Ni/CeO*_x_*, Co/CeO*_x_*, and Ni‐Co/CeO*_x_*. Reproduced with permission Ref. [132]. (C) Potential‐dependent in situ Raman contour maps of Cu and Ni(OH)*_x_*/Cu obtained during eNO_3_^−^RR in the potential range from 0.2 to −0.3 V versus reversible hydrogen electrode (vs. RHE). Reproduced with permission Ref. [101]. (D) Correlation between log_10_ (TOF, s^−1^) with weak and medium + strong acid sites. Reproduced with permission Ref. [80]. (E) SEM image of Cu_10_Fe_1_‐CFP. Reproduced with permission Ref. [103].

The synergistic effect between plasma‐activated N_2_ and catalyst surface plays a crucial role in the efficiency improvement of NH_3_ synthesis. This synergistic mechanism significantly reduces the high activation energy barrier of traditional thermal catalytic processes, thereby providing the core driving force to achieve efficient NH_3_ synthesis at ambient conditions. Ma et al. [131]. used a porous platinum (Pt) film deposited on the yttria‐stabilized zirconia tubes as a catalyst in this respect. The results demonstrate that the high‐density step sites and barrier‐free adsorption channels offered by the porous Pt membrane and its surface respectively make the N radicals generated by the RF discharge plasma easier to dissociate and adsorb. At this time, the energy barrier of N_2_ dissociation at the step sites becomes the core site for plasma activation of N_2_, as illustrated in Figure 12A. As a result, the plasma activates N_2_ to produce highly active species, and the catalyst exposes defect sites to lower the reaction energy barrier. In addition, the efficient N_2_ fixation can be achieved at room temperature, by optimizing the nanostructure of the catalyst to further increase the interaction interface. Luo et al. [132]. also developed a Ni‐Co/CeO*_x_* catalyst with atomic‐level dispersion of Ni and Co on the surface of CeO_2_ through the hydrothermal method. Among them, the Ni single atoms may reduce the formation energy of oxygen vacancies, causing the enrichment of oxygen vacancies (Figure 12B), while the Co single atoms can strengthen the adsorption of ·H. At the same time, the oxygen vacancies also provide an unsaturated coordination environment to reduce the ·H adsorption energy. The catalyst utilizes oxygen vacancy‐single atom dual active sites to synergistically regulate the highly active free radicals provided by the plasma, ultimately achieving a NH_3_ yield of 94.3 µmol g^−1^ h^−1^ with a high selectivity of ∼100%. Furthermore, it is demonstrated that the free radicals synthesize NH_3_ through the E‐R mechanism rather than the traditional L‐H mechanism. In the existing pNOR‐eNO*_x_* ^−^RR system, the H_2_O dissociation rate of Cu catalyst is relatively slow in alkaline environment, resulting in insufficient supply of adsorbed H_2_. For this reason, Liu et al. [101]. proposed a unique Ni(OH)_x_/Cu catalyst, through depositing amorphous Ni(OH)_x_ on the Cu surface. The Ni(OH)*_x_*/Cu interface may attract hydrated cations in the electrolyte via the non‐covalent interactions, thereby forming a local high‐concentration cation environment. The enriched cations further weaken the O─H bonds of interfacial H_2_O molecules, decrease the energy barrier of H_2_O dissociation, and accelerate the generation of adsorbed H_2_ species, thus providing sufficient proton source for the NO*_x_* ^−^RR. During the reduction reaction processes, the spark discharge non‐thermal plasma can oxidize the N_2_ in the air to NO*_x_*, while the latter is further converted into the NO*_x_* ^−^ with the KOH solution. The interfacial synergy between amorphous Ni(OH)*_x_* and Cu may also optimize the local micro‐environment through electronic modulation, thereby realizing the adsorption of NO*_x_* ^−^ on the Cu surface, reducing the energy barrier towards hydrogenation step of key intermediates, and inhibiting the formation of side reactions. This work achieved the series connection of non‐thermal plasma and interfacial H_2_O dissociation‐enhanced electrocatalyst (Figure 12C) for the first time, paving a new path for highly selective NH_3_ synthesis. Besides, Wang et al. [80]. synthesized an Al_2_O_3_‐supported transition‐metal catalyst, which achieved the activation of N_2_ to synthesize NH_3_ at room temperature, by regulating the chemical properties of metal surface, especially the distribution of acid site intensity, and coupling with non‐thermal plasma. The results show that the weak acid sites on the catalyst surface are positively correlated with the NH_3_ synthesis rate (Figure 12D). Among different catalysts, the Ni/Al_2_O_3_ has higher concentration of weak acid sites compared with the Cu/Al_2_O_3_ and Fe/Al_2_O_3_, and its synthesis rate is more than twice that of pure Al_2_O_3_. This is because weak acid sites promote the formation as well as conversion of NH_2_ intermediates, and accelerate the desorption of NH_3_ simultaneously, while the strong acid sites lead to excessive adsorption of NH_3_, thereby hindering the release of active sites. The work also reveals that surface chemical properties rather than physical structure dominate the plasma‐catalytic synergistic effect. Although this new technology effectively avoids the high energy consumption problem of traditional H‐B process, its plasma‐catalytic synergistic mechanism still needs to be further optimized for improving energy efficiency. Liu et al. [103]. also prepared the highly dispersed Cu‐Fe particles by the instantaneous thermal shock of Joule heating for NH_3_ synthesis at ambient temperature and pressure, using the soluble nitrates produced by plasma activation of N_2_ and O_2_ in the air as the nitrogen source. The instantaneous high temperature and ultrafast cooling process can suppress the separation of alloy phases, thereby forming a homogeneous solid solution. As for the carbon fiber paper (CFP), it may accelerate the electron transfer, and prevent nanoparticles sintering through the confinement effect. Therefore, its uniformly dispersed Cu‐Fe nanoparticles can expose more active sites than the naked counterparts (Figure 12E). Additionally, the free‐standing catalyst can also inhibit the occurrence of competitive hydrogen evolution reaction, optimize the rate‐controlling step (NO_3_^−^ → NO_2_^−^), accelerate mass transfer, and reduce intermediate product blockage. This work thus provides new ideas for the synthesis of green NH_3_.

In the plasma‐activated N_2_ system, optimizing plasma parameters plays a key role in increasing efficiency of NH_3_ synthesis. The results of laser‐induced plasma studies confirm that unfocus length (UL) of laser significantly affects the plasma intensity and NH_3_ yield. For example, when the UL is in the range of 0–1 mm, the plasma intensity is relatively high, with a NH_3_ yield exceeding 28 µmol min^−1^. However, the plasma weakens, and causes a significant decrease in NH_3_ yield (∼5.5 µmol min^−1^), as the UL >2 mm [110]. For the DBD, nitrogen‐rich atmosphere (N_2_:H_2_ = 3:1) is conducive to the dissociation of N_2_ as the rate‐controlling step, thereby improving the synthesis efficiency [44]. Increasing the driving frequency (52–65 kHz) can enlarge the micro‐discharge density, thereby promoting N_2_ dissociation and raising NH_3_ yield, but if the frequency is too high (e.g., 68 kHz), the yield may also decrease slightly, due to the reaction equilibrium limitation [133]. In the double dielectric barrier discharge process, the highest NH_3_ yield can be achieved at low power (5–6 W) and 450 K over a Ni catalyst. Nevertheless, excessive power will accelerate the decomposition of NH_3_, because of local overheating, thereby reducing synthesis efficiency [134]. Based on above considerations, it can be inferred that precise control of plasma parameters is crucial to optimizing the plasma‐activated NH_3_ synthesis process. This requires striking a balance between enhancing key steps and avoiding disadvantages.

The synergistic effect of plasma and catalyst can activate N_2_ to generate highly active free radicals. Combined with the synergistic interaction between the catalyst surfaces, it significantly reduces the activation energy of N_2_ at room temperature and pressure, breaking through the thermodynamic limitations of traditional thermal catalytic pathways. In the meantime, the precise control of plasma parameters is the key to enhancing reaction kinetics and suppressing side reactions. Thus, the plasma coupled with catalytic technology offers a technical paradigm for the development of new catalysts in green NH_3_ synthesis, and the exploration of synergistic optimization method of plasma and reactor structure in the future.

## 5 Energy Consumption Analysis of Plasma‐Assisted NH_3_ Synthesis

**FIGURE 13:**
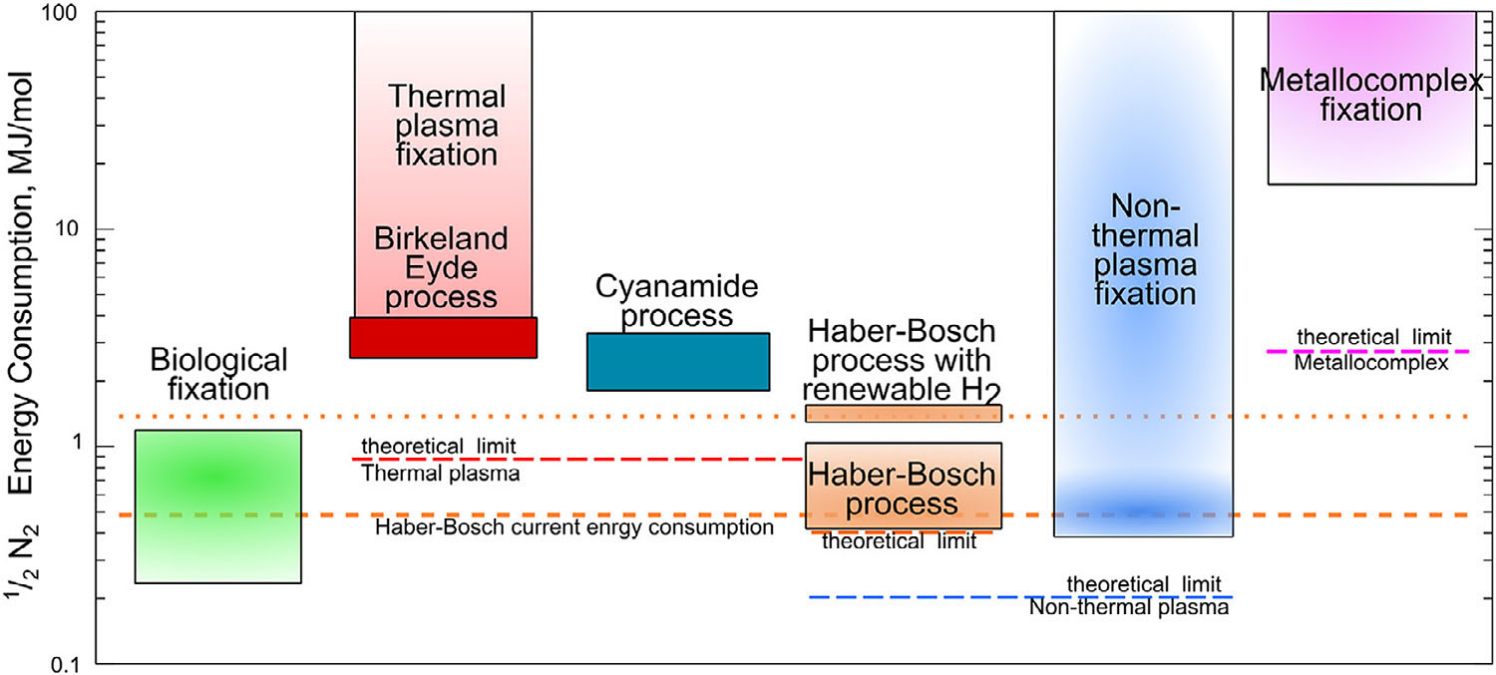
A comparison for the energy consumption of N_2_ fixation with different methods. Reproduced with permission Ref. [35].

Energy consumption is a comprehensive indicator that reflects the energy utilization rate of a process, and is also a summary indicator that ultimately reflects the economic benefits of industrial NH_3_ synthesis [135]. Therefore, it is of interest to focus on the energy consumption of plasma‐activated N_2_ fixation. At present, the energy consumption of traditional H‐B process has reached a bottleneck, that is, 0.48 MJ $\mathrm{mo}l_{NH_{3}}$ ^−1^ [136]. Contrarily, the energy consumption of plasma‐assisted NH_3_ synthesis is still expected to be further reduced [35]. As illustrated in Figure 13, although the H‐B process still owns the lowest energy consumption, it is already close to the theoretical limit. In comparison, the non‐thermal plasma‐assisted NH_3_ synthesis has a larger room for energy efficiency improvement than the H‐B process, showing a greater competitive advantage. In particular, the electrochemical NH_3_ synthesis technology has attracted widespread attention, with the rapid development of renewable energy and the rise of distributed energy system, because it could operate under mild conditions and be coupled with green electricity resources. Nevertheless, the energy consumption of H‐B process is far lower than that of most electrochemical synthesis pathways, resulting from nearly a century of process optimizations and scale economies. For example, the energy consumption of electrochemical method based on the PEM technology is as large as 98.1–220.7 MJ mol^−1^, while its FE is only 0.16%–0.36%, reflecting that the NRR faces severe kinetic barriers and fierce competition from side reactions [137]. At present, some electrochemical pathways have made significant progress in energy consumption, and shown great development potential. In this regard, the energy consumption of mixed conducting electrolyte route is about 7.97–69.89 MJ mol^−1^, which is far superior to that of PEM system [138]. More impressively, the alkaline exchange membrane (AEM) system further reduces the energy consumption of electrochemical NH_3_ synthesis to 0.858–31.88 MJ mol^−1^ [139]. However, the limit still exists, that is, although the FE of AEM technology can reach 41% in the initial stage, it quickly declines to single‐digit level within a few hours. Such rapid performance decay reveals that the electrochemical NH_3_ synthesis still faces huge challenges in the long‐term stability. In the future, it is necessary to maintain high FE while taking into account the stability of electrochemical NH_3_ synthesis, as a powerful alternative to the conventional H‐B process. A brief summary for the energy consumption of plasma‐enhanced N_2_ fixation is listed in Table 4. In general, the plasma‐based N_2_ fixation can be described in the following two pathways.

**TABLE 4:** A summary for the energy consumption of reported plasma‐catalyzed NH_3_ synthesis.

Pathway	Plasma type	Catalyst	Gas	Energy consumption	Energy consumption	Ref.
				MJ $\mathrm{mo}l_{NO_{x}}$ ^−1^	MJ $\mathrm{mo}l_{NH_{3}}$ ^−1^	
H‐B Plasma	/	Fe	N_2_, H_2_	/	0.48	[136]
	DBD	/	N_2_, H_2_	/	59	[104]
	DBD	Ni/LaOF	N_2_, H_2_	/	22.6	[51]
	DBD	Ni/Al_2_O_3_	N_2_, H_2_	/	20.82	[80]
	RF	Ni‐MOF‐74	N_2_, H_2_	/	265	[54]
	RF	Au wire	N_2_, H_2_	/	322.7	[106]
	MW	/	N_2_, H_2_	/	1532.8	[61]
Electrocatalysis	/	Pt	N_2_, H_2_	/	98.1–220.7	[137]
	/	Perovskite	Air	/	7.97–69.89	[138]
	/	Pt, Fe, Ni, FeNi	Air	/	0.858–31.88	[139]
Plasma electrolysis	CD	Cu_2_O nanowire	Air	8.604	14.076	[63]
	Jet	/	N_2_	/	138.96	[107]
	DBD	/	Air	1.06	15.49	[37]
	Jet	Co_3_O_4_	Air	40.248	>40.248	[62]
	GA	Co SAs/N‐C	N_2_/O_2_	2.4	3.18	[108]
	GAD + MW‐PM	PtCu NP	Air	/	33.78	[109]

The pathway 1 is N_2_→NH_3_. Over the past decade, researchers have been working on the plasma‐assisted reduction of N_2_ by H_2_ using diverse catalysts for continuous improvement in production efficiency and energy consumption of NH_3_ synthesis. Representatively, Patil et al. [69]. screened 16 different transition metals as well as oxides, and discussed their effects on the DBD plasma‐catalyzed synthesis of NH_3_ in detail. By optimizing the feed ratio, specific energy input, reaction temperature, metal loading and gas flow rate, a low energy consumption of 32 MJ $\mathrm{mo}l_{NH_{3}}$ ^−1^ was achieved. The results demonstrated that the matching between plasma discharge parameter and catalyst was also very critical, besides the catalyst screening. Kim et al. [140]. happen to be coincident with the Ptail's findings, and realized a low energy consumption of 28.01 MJ $\mathrm{mo}l_{NH_{3}}$ ^−1^ in the nanosecond pulsed‐filled‐bed reactor with a Ru(2)‐Mg(5)/γ‐Al_2_O_3_ catalyst accompanied by a feed ratio of N_2_:H_2_ = 4:1 at 300°C and atmospheric pressure. Through the understanding of conventional H‐B process and above‐mentioned plasma routes, it can be known that the one‐step synthesis of NH_3_ using N_2_ and H_2_ as feedstock involves an additional H_2_ production step, and thus requires a lot of energy consumption. In contrast, non‐hydrogen reduction N_2_ fixation technology not only avoids this energy‐intensive step, but also simplifies the synthetic procedure.

The pathway 2 is N_2_→NO*_X_* ^−^→NH_3_. In addition to the direct one‐step synthesis of NH_3_ via the NRR with H_2_, N_2_ can also be oxidized firstly to reactive NO*_x_* ^−^ species by O_2_, and then further reduced to NH_3_. In this regard, Liang et al. [141]. proposed a cascade connected N_2_ fixation strategy with the DBD plasma oxidation and electrocatalytic reduction (pNF‐eNO*_x_* ^−^RR), which could complete the NH_3_ synthesis process through a two‐step reactions. For instance, N_2_ is oxidized to be NO*_x_* ^−^ by the plasma, while the latter is subsequently absorbed by the KOH solution, and electro‐reduced to NH_3_. This process may achieve a high NH_3_ yield of 16.21 mg h^−1^ with an energy efficiency of 2.08%, and a total energy consumption of 18.36 MJ $\mathrm{mo}l_{NH_{3}}$ ^−1^ that is much lower than that of the plasma‐activated proton‐conducting solid‐oxide electrolyzer (605 MJ $\mathrm{mo}l_{NH_{3}}$ ^−1^) [99]. Zheng et al. [128]. also connected the plasma in series with a dual electrocatalytic electrolyzer, and developed a matching catalyst, that is, the defective N‐doped molybdenum sulfide nanosheets loaded on vertical graphene arrays (N‐MoS_2_/VGs), thus achieving a low energy consumption of 2.4 MJ $\mathrm{mo}l_{NH_{3}}$ ^−1^. Although their energy consumption is higher than that of the traditional H‐B process, the pNF‐eNO*_x_* ^−^RR system can still provide an alternative route for the development of decentralized NH_3_ synthesis industry, considering that the H‐B process is only economically feasible in large‐scale production.

Based on the understanding of development status in plasma‐catalyzed NH_3_ synthesis technology, one may infer that although its energy consumption is inferior to that of the H‐B industry, it has more obvious advantages in compatibility with renewable energy, such as solar, wind and tidal resources [142, 143, 144, 145]. Meanwhile, its operating conditions are also milder than those of conventional method. Therefore, it may be a feasible and competitive alterative with good development and application potential for remote areas or small‐scale, decentralized NH_3_ production.

## 6 Conclusions and Outlook

In summary, we review the current status of research on the plasma‐enhanced NH_3_ synthesis, while comprehensively sorting out the problems existing in the synthetic process. Up to date, a variety of plasmas have been used, showing broad application prospects in NH_3_ synthesis. The future research focus of plasma‐enhanced NH_3_ synthesis will mainly include the following aspects.

1. The optimization of reaction system: the plasma activation process consumes a large amount of energy, and produces active substances with a short lifetime, thus it is necessary to optimize the reactor design for improving the activation efficiency of plasma on N_2_. Currently, the DBD plasma is the most commonly used discharge strategy in the NH_3_ synthesis process, and correspondingly various DBD reactors, including fixed bed, packed bed, and fluidized, etc., are developed. Optimizing key parameters, such as electrode type and shape, discharge gap and type, dielectric constant, and gas dilution, can effectively reduce the energy cost of NH_3_ synthesis. In addition, the gas flow field inside the reaction device must be rationally designed, so that the raw materials can be evenly distributed in the plasma, thereby increasing the probability of collision between the reaction gas and high‐energy particles. Installing the gas distributor along with optimizing the shape as well as size of the reactor can also promote gas turbulence, and thus enhance mass transfer. Based on these measures, the active substances produced by N_2_ activation can be fully utilized, and meanwhile the loss of energy and substances may be effectively avoided, too.
2. The development of highly synergistically active catalysts, and the usage of aprotic solvent are key to promoting N_2_ dissociation and adsorption reactions of surface‐active species as well. Among them, the catalyst selection in plasma‐assisted NH_3_ synthesis, including catalyst precursor, support and additive, is particularly important. First, the designed catalyst should be able to promote the generation of active N_2_ and H_2_ species in the plasma, and accelerate their reaction, thereby increasing the NH_3_ yield. For instance, the catalyst surface should have a suitable electronic structure to facilitate the adsorption, activation and conversion of active species. Secondly, one of the bottlenecks hindering the industrialization of plasma‐enhanced NH_3_ synthesis is the poor interaction between plasma and catalyst, which has not been fully resolved. Currently, the combination of Co‐Ni/MOF‐74 catalyst with DBD plasma has only achieved a NH_3_ yield of ∼43.48 µmol g^−1^ min^−1^ at ambient temperature and pressure. This requires in‐depth research on their synergistic mechanism, and continuous optimization of the catalyst's property and structure based on the plasma discharge mode and equipment parameters, etc. These help to achieve a successful match between catalyst and plasma, thereby improving the energy efficiency and production rate of NH_3_ synthesis. Therefore, it is necessary to explore more advanced in situ characterization techniques, such as infrared, synchrotron radiation X‐ray absorption, laser‐induced fluorescence and laser‐scattering spectroscopies, to monitor the activation degree of N_2_, the generation and consumption of intermediates, and the synthesis rate of NH_3_. Real‐time analysis of the above parameters is conducive to investigating the influence of catalyst on the physicochemical property of plasma, thereby revealing the synergistic mechanism of plasma‐catalyzed NH_3_ synthesis.
3. In‐depth study of the NRR mechanism is of great significance to improving NH_3_ yield too, which can offer theoretical guide for the selection of raw materials and the optimization of reaction conditions. The investigation of reaction mechanism is helpful to understand all aspects of the NRR from its source. Specifically, there is still controversy over the specific pathway and rate‐determining step of NRR process. At the same time, the synergistic reaction mechanism of plasma catalysis needs further in‐depth study. In the future, researchers should establish a more accurate kinetic model of plasma‐activated N_2_ fixation. After that, the reaction mechanism of plasma‐activated NRR can be investigated in depth by using quantum chemical calculations and molecular dynamics simulations, including the N_2_ dissociation, free radical generation, intermediate product, and NH_3_ desorption, etc. This is of vital importance in guiding specific experiments.
4. Combining plasma with other technologies, including electrocatalysis, photocatalysis, and thermocatalysis, etc., can not only give full play to the advantages of each technology, but also overcome the limitations of a single technology. For example, a high NH_3_ yield of 2329.4 µmol h^−1^ cm^−2^ with a FE of 96.08% can be achieved in the electrocatalytic system, through the integration of plasma with Co_3_O_4_ catalyst. But as one of very important means to significantly reduce the energy consumption, and elevate the NRR efficiency, coupling of plasma technology and other catalytic technologies still has much room for improvement. This also requires continuous pursuit of new N_2_ fixation technologies with high economic benefit and efficiency. In this regard, it is recommended to closely combine the plasma reactor with other catalytic reactors by constructing an integrated reaction system, thereby reducing energy and product losses in the intermediate transmission process. Correspondingly, theoretical studies on the plasma‐catalyzed coupling reactions should also be carried out. For example, the formation mechanism and charge transfer process of active species on the electrode surface during the plasma‐assisted electrocatalysis, as well as the energy transformation and synergistic mechanism between different plasma and photo/thermocatalysis might be worth further investigation. The above researches can offer a theoretical basis for the development of new and efficient integration technologies, thereby promoting the industrialization of plasma‐catalyzed NH_3_ synthesis.

## Conflicts of Interest

The authors declare no conflicts of interest.

## Data Availability

The data that support the findings of this study are available on request from the corresponding author. The data are not publicly available due to privacy or ethical restrictions.
